# Targeting ROAM1 with UDP-GlcNAc nanosheets selective activates lysosomal AMPK to resolve metabolic dysfunction-associated steatotic liver disease

**DOI:** 10.1016/j.bioactmat.2026.06.047

**Published:** 2026-07-12

**Authors:** Jianxi Zhu, Tuanwei Sun, Fuxin Wei, Dong Wang, Feng Liu, Dongming Chen, Xian Liu, Chuanle Wang, Shengcheng Deng, Yue Yang, Yuanyan Xiong, Hongfei Xiang, Jiyu Li, Songbo Li, Wenbin Ma, Songyang Zhou

**Affiliations:** a Shenzhen Key Laboratory of Bone Tissue Repair and Translational Research, Department of Orthopaedic Surgery, The Seventh Affiliated Hospital of Sun Yat-sen University, Shenzhen, 518107, China; b MOE Key Laboratory of Gene Function and Regulation and Guangzhou Key Laboratory of Healthy Aging Research, School of Life Sciences, Sun Yat-sen University, Guangzhou, 510275, China; c Innovative Center of Health, Longevity and Synthetic Biology, Hainan Academy of Medical Sciences, Hainan Medical University, Haikou, 571199, China; d Sun Yat-sen Memorial Hospital, Sun Yat-sen University, Guangzhou, 510120, China; e Division of Orthopaedic Traumatology and Microsurgery, Center for Orthopaedic Medicine, The Seventh Affiliated Hospital of Sun Yat-sen University, Shenzhen, 518107, China; f Department of Orthopedics, The Affiliated Hospital of Qingdao University, Qingdao University, Qingdao, 266003, China; g School of Biomedical Engineering, Sun Yat-sen University, Shenzhen, 518107, China; h The Tenth Affiliated Hospital, Southern Medical University (Dongguan People's Hospital), Dongguan, 523059, China; i Dongguan Key Laboratory of Basic, Clinical and Digital Research on Common Orthopedic Diseases, Dongguan, 523059, China

## Abstract

Metabolic dysfunction-associated steatotic liver disease (MASLD) is a prevalent chronic liver disease with limited treatment options. Although AMP-activated protein kinase (AMPK) has been implicated in multiple pathological processes and represents a highly promising therapeutic target for MASLD, the clinical efficacy of AMPK activators has been unsatisfactory, possibly due to overly abundant substrate and the complex activation mechanisms of AMPK. Recent work on lysosome-specific activation of AMPK has revealed a preference for metabolic substrate activation, highlighting it as a potential target for precision therapy of MASLD. Here, through a bimolecular fluorescence complementation (BiFC)-based protein interaction screen, we identify the nucleotide-sugar transporter ROAM1 (renamed from SLC35F6) as an AMPKβ-interacting protein that localizes to the lysosome and negatively regulates AMPK activity. Silencing ROAM1 in mouse liver and muscle elevates basal AMPK activity and induces a transcriptional state that inhibits lipid synthesis. UDP-GlcNAc is the ligand of ROAM1 and activates lysosomal AMPK through the ROAM1-AMPKbeta axis to primarily regulate lipid metabolism. To evaluate the therapeutic effect of this pathway on MASLD, we engineered a magnesium-coordinated UDP-GlcNAc nanosheet (MgUGN) for efficient *in vivo* delivery. MgUGN treatment improves lipid metabolism and reduces hepatic steatosis in metabolic disease models, and mitigates liver damage in acute injury models by decreasing inflammation. These findings identify the UDP-GlcNAc-ROAM1-AMPK axis as a regulator of hepatic lipid metabolism and introduce MgUGN as a novel compartment-specific AMPK activator for liver disease.

## Highlights

• ROAM1 is a lysosomal AMPK-interacting protein that negatively regulates basal AMPK activity and lipid synthesis.
• UDP-GlcNAc activates lysosomal AMPK via the ROAM1-AMPKβ axis to selectively control lipid metabolism.
• Magnesium-coordinated UDP-GlcNAC nanosheets overcome the poor cellular uptake and enable sustained AMPK activation.
• MgUGN alleviates hepatic steatosis, inflammation, and injury in both chronic MASLD and acute liver injury models.

## Graphical abstract

Nanosheet-mediated UDP-GlcNAc delivery activates AMPK via the lysosomal transporter ROAM1, protecting against liver injury.

**Figure:**
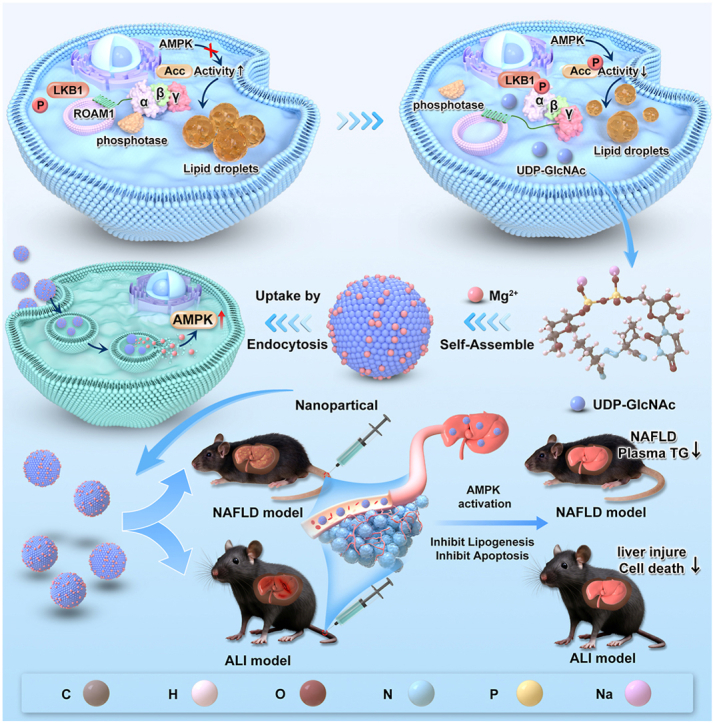

## 1 Introduction

Metabolic dysfunction-associated steatotic liver disease (MASLD), formerly known as non-alcoholic fatty liver disease (NAFLD), has become a leading cause of chronic liver disease worldwide, affecting approximately 38% of adults and an even higher proportion—65%—of patients with type 2 diabetes mellitus [1,2]. Regarding its natural history, approximately 7–12% of individuals with MASLD progress to cirrhosis or end-stage liver disease over 7–20 years. This condition is also closely associated with cardiovascular disease and extrahepatic malignancies [3]. The pathogenesis of MASLD is complex and multifactorial, involving hepatic steatosis, insulin resistance, lipotoxicity, oxidative stress, endoplasmic reticulum stress, and chronic low-grade inflammation [4,5]. Accumulating evidence indicates that metabolic inflammation and impaired energy homeostasis are key drivers of disease progression from simple steatosis to metabolic dysfunction-associated steatohepatitis (MASH) and cirrhosis [6]. To date, the lack of effective pharmacotherapies underscores the urgent need for novel therapeutic targets and strategies to improve patient outcomes.

Among the key regulators of cellular metabolism, AMP-activated protein kinase (AMPK) has emerged as a particularly promising target for MASLD intervention [7,8]. AMPK serves as a central sensor of cellular energy status, and its activity is notably reduced in the livers of patients and animal models with MASLD [7,8]. Restoration of AMPK activity has been shown to ameliorate multiple pathological features of MASLD, including hepatic steatosis, inflammation, and fibrosis [7,8]. AMPK functions as a heterotrimeric complex composed of a catalytic α subunit and regulatory β and γ subunits [9]. The β subunits serve as scaffolds, facilitating complex assembly through their C-terminal domains [10]. The γ subunit adopts distinct conformations based on cellular AMP/ADP/ATP levels [11]. Under energy stress, an elevated AMP/ATP ratio promotes AMP binding to AMPKγ, driving a conformational change that enables the upstream kinase LKB1 to phosphorylate Thr172 on the α subunit, thereby increasing AMPK activity [[12], [13], [14], [15], [16]]. Once activated, AMPK restores energy balance by promoting catabolic pathways (e.g., fatty acid oxidation, glycolysis, and autophagy) while inhibiting anabolic processes (e.g., lipogenesis, gluconeogenesis, and protein synthesis) [17].

In the context of MASLD, AMPK activation exerts multiple beneficial effects: it directly phosphorylates and inactivates acetyl-CoA carboxylase (ACC), thereby reducing malonyl-CoA levels and promoting fatty acid oxidation [18]; it phosphorylates and inhibits sterol regulatory element-binding protein 1c (SREBP1c), a master transcription factor controlling de novo lipogenesis, thereby reducing hepatic triglyceride accumulation [19]; and it suppresses inflammatory responses via inhibiting NF-κB signaling [20]. Furthermore, AMPK activation improves mitochondrial function and reduces oxidative stress, contributing to the attenuation of hepatocyte injury and fibrosis [21]. These multifaceted actions make AMPK an attractive therapeutic target for MASLD. Importantly, AMPK activity is significantly downregulated in MASLD, and its activation ameliorates hepatic steatosis, inflammation, and fibrosis [7]. However, existing AMPK activators, including A769662 and AICAR, have failed to achieve satisfactory clinical efficacy for MASLD despite promising preclinical results, partly due to their limited potency, poor bioavailability, off-target effects, and the complex activation mechanisms of AMPK that involve numerous functionally distinct substrates [22]. Therefore, deeper insights into AMPK regulatory mechanisms should greatly facilitate the development of precise AMPK activation strategies to treat diseases such as MASLD.

Recent studies have revealed that compartment-specific activation of AMPK enables selective phosphorylation of downstream substrates. For instance, lysosomal activation of AMPK preferentially targets metabolic-related substrates, thereby regulating cellular metabolism [23,24]. Under starvation, Axin helps recruit AMPK to the lysosomal surface, facilitating its interaction with the lysosomal v-ATPase, Ragulator complex, and LKB1, which enhances Thr172 phosphorylation and subsequent AMPK activation [25]. Lysosomal AMPK activation increases phosphorylation of substrates such as sterol regulatory element binding transcription factor 1 (SREBP1c), TSC complex subunit 2 (TSC2), acetyl-CoA carboxylase1 (ACC1), and regulatory associated protein of MTOR complex 1 (Raptor), but not mitochondrial fission factor (MFF) or acetyl-CoA carboxylase1 (ACC2), demonstrating substrate selectivity [23,24]. These spatially restricted activation mechanisms suggest that the subcellular localization of AMPK determines its substrate preference and functional output, offering new opportunities for developing precision therapies for metabolic diseases including MASLD [26].

However, the understanding of AMPK regulation network at the lysosome remains incomplete for development precisely activator for lysosome AMPK activation. For example, lysosomal AMPK activation can occur in the absence of dramatic energy depletion [27], suggesting the existence of additional sensors for local nutrient cues. Moreover, the endogenous factors that limit lysosomal AMPK activation are still poorly understood. The β subunits of AMPK are critical for its lysosomal localization. Post-translational modifications such as N-myristoylation at residue Gly2 on the β subunits are essential for AMPK membrane association and for its activation in response to metabolic stress [[28], [29], [30]]. Substituting Gly2 with Ala disrupts this modification, impairing AMPK lysosomal localization and diminishing AMPK activation in an AMP-independent manner under glucose starvation [30].

In this study, we systematically analyzed the interaction networks of AMPK β subunits in an effort to discover new upstream components of AMPK signaling and to identify suitable therapeutic targets that can precisely regulate lysosomal AMPK activation for the treatment of MASLD. We took advantage of the bimolecular fluorescence complementation (BiFC) method to screen for novel interacting proteins of AMPK β1 and β2 subunits. The screen identified the nucleotide-sugar transporter SLC35F6/ROAM1 as a lysosomal protein that interacts with AMPK and negatively regulates its activity both in cells and *in vivo*. We showed that increased levels of UDP-GlcNAc, a cargo of the SLC35 family transporters, activated AMPK in a SLC35F6/ROAM1-dependent manner. The UDP-GlcNAc-ROAM1-AMPK signaling axis preferentially phosphorylates metabolic-related substrates, particularly suppressing cellular lipid synthesis. These results demonstrate for the first time that UDP-GlcNAc functions as an AMPK activator that preferentially regulates lipid metabolism. In mouse liver disease models, a UDP-GlcNAc-derived nanosheet exerts both metabolic and hepatoprotective effects, suggesting dual therapeutic potentials for UDP-GlcNAc and for targeting SLC35F6/ROAM1. Together, these findings reveal a previously unrecognized mechanisms for lysosomal AMPK activation and a nanodrug that exhibits potent therapeutic efficacy against MASLD.

## 2 Results

### 2.1 Lysosome protein ROAM1 interacts with AMPKβ and directly inhibits AMPK activity

**Fig. 1:**
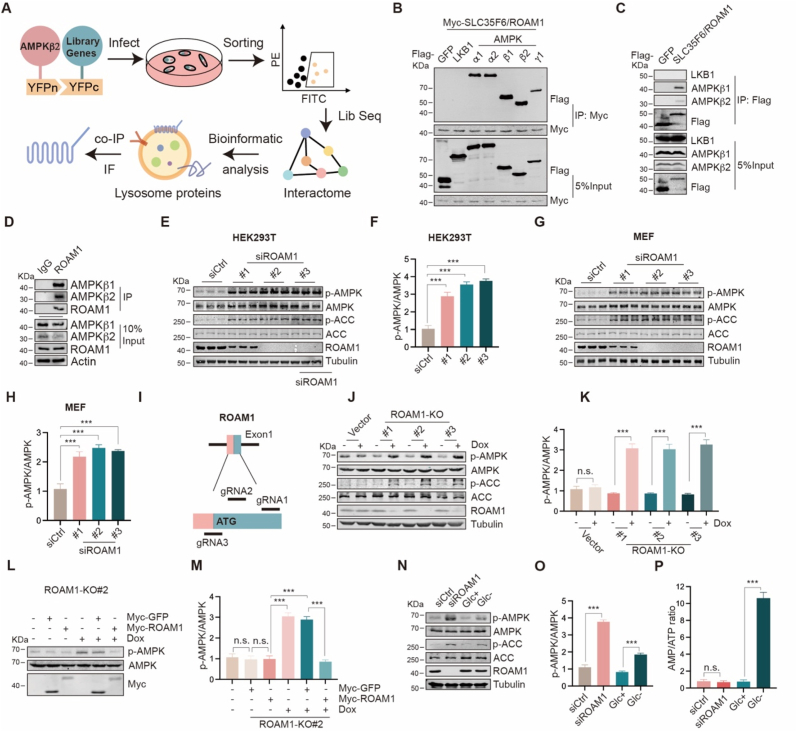
The lysosomal protein SLC35F6/ROAM1 interacts with AMPK and suppresses AMPK activ**ation. (A)** For the genome-wide BiFC screen, we tagged AMPKβ1 and AMPKβ2 with the N-terminal half of Venus YFP (YFPn) and ectopically expressed the proteins in HTC75 cells stably expressing the human ORFome library tagged with the C-terminal half of YFP (YFPc). Interactions between the bait and prey library proteins brings the two YFP fragments together for co-folding and fluorescence complementation, which can be detected by a flow cytometer. YFP + cells enriched by successive rounds of FACS sorting were then harvested for RT-PCR and next-generation sequencing to identify potential AMPKβ-interacting proteins. Proteins predicted to localize to lysosomes were next selected for co-immunoprecipitation (co-IP) and indirect immunofluorescence (IF) assays. **(B)** Myc-tagged SLC35F6/ROAM1 was co-expressed with Flag-tagged AMPKα1, AMPKα2, AMPKβ1, AMPKβ2, or AMPKγ1 in HEK293T cells. Cells were then harvested for anti-Myc IP and western blotting (WB) as indicated. Flag-tagged GFP and LKB1 served as negative controls. **(C)** HEK293T cells expressing Flag-tagged SLC35F6/ROAM1 were harvested for anti-Flag IP and WB as indicated. Flag-tagged GFP served as a negative control. **(D)** Endogenous interaction between ROAM1 and AMPKβ1/β2. Endogenous ROAM1 was immunoprecipitated from mouse liver tissue lysates using a specific anti-ROAM1 antibody. The immunocomplexes were captured by protein A/G agarose beads and analyzed by Western blotting with the indicated antibodies. Normal rabbit IgG served as a negative control. **(E**–**H)** HEK293T (E) and MEF (G) cells expressing three different siRNAs against ROAM1 (siROAM1) were collected three days after transfection for WB as indicated. A scramble siRNA was used as control (siCtrl). p-AMPK, T172 phosphorylation. p-ACC, S79 phosphorylation. The ratios of p-AMPK/AMPK are quantified in (F) and (H) as mean ± SD. ∗∗∗p < 0.001by one-way ANOVA, n = 3. **(I**–**K)** Three gRNAs targeting different regions of SLC35F6/ROAM1 exon 1 (I) were individually introduced into HEK293T cells expressing doxycycline (Dox)-inducible Cas9, in which Dox addition should induce Cas9 expression and subsequent ROAM1 knockout (KO). The cells were cultured in the presence or absence of Dox for three days and then harvested for WB (J) and p-AMPK/AMPK quantification (K) as indicated. Statistical analysis results are shown as mean ± SD. ∗∗∗p < 0.001 by one-way ANOVA, n = 3. n.s., not significant. **(L**–**M)** ROAM1-KO #2 cell line from (J) that also ectopically expressed Myc-tagged ROAM1 were cultured ± Dox before WB (L) and p-AMPK quantification (M). Myc-GFP served as a negative control. Statistical analysis results are shown as mean ± SD; ∗∗∗p < 0.001by one-way ANOVA, n = 3. n.s., not significant. **(N**–**P)** HEK293T cells treated with siROAM1 (#3) or glucose starvation (Glc-) were collected for WB (N) and AMP/ATP detection by LC-MS (P). Quantification of p-AMPK/AMPK is shown in (M). Statistical analysis results are shown as mean ± SD; ∗∗∗p < 0.001 by one-way ANOVA, n = 3. n.s., not significant. Cells expressing siCtrl or grown in glucose-supplemented media (Glc+) served as controls.

Given that AMPKβ plays a key role in enabling AMPK heterotrimers to associate with the lysosomal membrane, we sought to identify its interacting proteins to better understand compartmentalized AMPK signaling. To this end, we used a genome-wide screening platform based on bimolecular fluorescence complementation screens (BiFC), which we developed to minimize complications from membrane-bridged non-specific interactions (Fig. 1A) [[31], [32], [33]]. We tagged AMPKβ1 and AMPKβ2 with the N-terminal half of Venus YFP (YFPn) and expressed them as baits in the HTC75 prey library cell line we had previously generated [32]. This cell line stably expresses approximately 18,000 genes from the human ORFeome library, each tagged with the C-terminal half of YFP (YFPc). Fluorescence complementation, indicative of prey-bait interaction, was detected by fluorescence-activated cell sorting (FACS). Once YFP-positive cells were enriched to a purity of >90% (Supplementary Fig. S1A–B), they were collected for PCR amplification and next-generation sequencing (Fig. 1A). Multiple known AMPKβ-interacting proteins that were represented in the library were identified from our screen (Table 1), including AMPKα1, AMPKγ1, AMPKγ2, PKM2, LKB1, MDM4, and TSC2 [[34], [35], [36]]. We then categorized all candidate proteins based on their known or predicted subcellular localization and selected those predicted to localize to lysosomes by the Human Protein Atlas (https://www.proteinatlas.org/) and UniProt (https://www.uniprot.org/). Among these, the solute carrier family member SLC35F6 contains a highly conserved lysosomal sorting signal motif (D/EXXXL)(Supplementary Fig. 1C), and has been reported to localize to lysosomes [37]. Indeed, Flag-SLC35F6 could co-localize with the lysosomal marker LAMP1 (Supplementary Fig. 1D). The solute carrier 35 (SLC35) family, a large group of evolutionarily conserved hydrophobic proteins with multiple transmembrane domains, are also known as nucleotide-sugar transporters (NSTs). When co-expressed, Myc-tagged SLC35F6 co-immunoprecipitated (co-IP) with each of the Flag-tagged AMPK subunits (α, β, or γ) (Fig. 1B). Similarly, Flag-SLC35F6 could co-IP with endogenous AMPK β1 and β2, but not endogenous LKB1 (Fig. 1C). The endogenous interaction between SLC35F6 and AMPKβ subunits was further confirmed by co-immunoprecipitation using specific antibodies against endogenous SLC35F6 and AMPKβ1/β2 in mouse liver tissue (Fig. 1D). Together, these results indicate that SLC35F6 is a previously unrecognized interacting partner of AMPKβ subunits on lysosomes. Because of its role, we refer to it as Regulator of AMPK at Membrane 1 (ROAM1) hereafter.

To determine whether ROAM1 regulates AMPK activation, we generated ROAM1 knockdown (KD) HEK293T cells using three distinct siRNAs and examined the phosphorylation of T172 in the activation loop of AMPKα, the primary readout of AMPK activation [38]. ROAM1 KD led to substantial depletion of ROAM1 proteins in these cells, and a concomitant increase in T172 phosphorylation without affecting total AMPKα protein levels (Fig. 1E–F). Acetyl-CoA carboxylase (ACC) is a key enzyme in fatty acid synthesis and a direct AMPK substrate. Phosphorylation of ACC at Ser79 serves as a widely used readout of AMPK activation. Following ROAM1 KD, ACC phosphorylation was also increased (Fig. 1E–F), consistent with ROAM1 as a negative regulator of AMPK. Similar results were also obtained in mouse embryonic fibroblasts (MEFs) and the hepatocellular carcinoma cell line HepG2 (Fig. 1G–H and Supplementary Fig. S1E–F). Activated AMPK is known to inhibit protein synthesis by downregulating mTOR signaling [35]. In line with ROAM1 negatively regulating AMPK activity, phosphorylation of S6K, a downstream mTORC1 target involved in protein synthesis, was dramatically reduced in ROAM1 KD HEK293T cells (Supplementary Fig. S1G and H) [39]. These data indicate that ROAM1 suppresses AMPK activation in a cell type-independent manner. To further validate our findings, we established doxycycline (Dox)-inducible ROAM1 knockout (KO) cell lines (Fig. 1I). ROAM1 KO upon Cas9 induction led to a marked increase in AMPK T172 and ACC S79 phosphorylation (Fig. 1J–K), a phenotype rescued by the ectopic expression of ROAM1 (Fig. 1L–M), supporting ROAM1's role in repressing AMPK activity.

AMPK is primarily activated under low-energy conditions, reflected by elevated AMP/ATP ratios, as occurs during starvation, mitochondrial dysfunction, or prolonged exercise [17]. As expected, glucose-starved (Glc-) cells exhibited a substantially elevated AMP/ATP ratio, as measured by LC-MS, along with elevated AMPK phosphorylation (Fig. 1N–P). In contrast, increased AMPK phosphorylation in ROAM1 KD cells was not accompanied by altered AMP/ATP ratios (Fig. 1N–P), suggesting a bypass of AMP sensing with ROAM1 KD-induced AMPK activation. To probe this possibility further, we generated HEK293T cells stably expressing the AMP-insensitive R531G mutant of the AMPKγ2 subunit [40]**,** which is unresponsive to the AMPK activator AICAR (Supplementary Fig. 1I–J). Notably, ROAM1 depletion in these cells still led to enhanced AMPK T172 phosphorylation (Supplementary Fig. 1I–J). These findings indicate that ROAM1 regulates AMPK activation independent of changes in AMP/ATP ratios. We also assessed mitochondrial membrane potential, which can activate AMPK when reduced, but observed no changes in MitoTracker fluorescence in ROAM1 KD cells (Supplementary Fig. S1K–L). Together, these results indicate that ROAM1 suppresses AMPK activity independently of AMP sensing and mitochondrial depolarization, highlighting its potential to precisely regulate lysosomal AMPK activity.

### 2.2 AMPK activation induced by ROAM1 downregulation decreases fat synthesis *in vitro* and *in vivo*

**Fig. 2:**
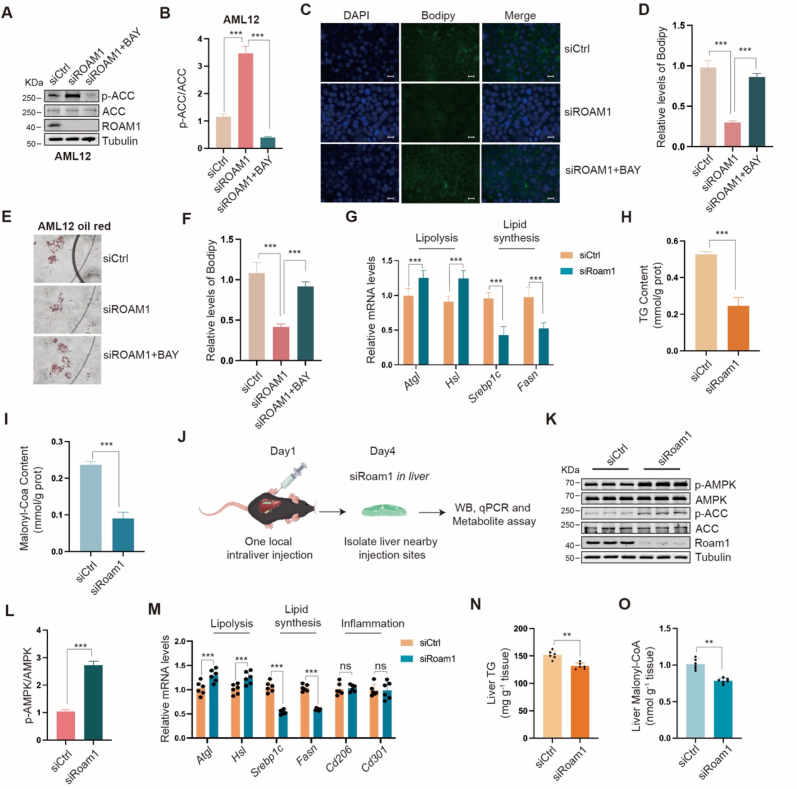
ROAM1 downregulation induces metabolic changes via activation of A**MPK. (A**–**B)** AML12 cells expressing siRNAs against ROAM1 (mouse siROAM1) were treated with or without BAY-3827 (40 μM) for 1 h three days after transfection. Cells were then collected for WB using the indicated antibodies (A) and p-ACC/ACC ratio quantification (B). Statistical analysis results are shown as mean ± SD; ∗∗∗p < 0.001 by one-way ANOVA, n = 3. siCtrl, a scramble siRNA. **(C**–**D)** Cells from (A) were stained with the BODIPY 493/503 dye (green) (C), which can stain neutral lipids and act as a tracer for oil and other nonpolar lipids. Hoechst 33342 (blue) was used to stain nuclear DNA (Scale bar: 10 μm). Fluorescence signals were quantified and plotted in (D) as mean ± SD (n = 3). Statistical significance was calculated using the Student's t-test. ∗∗∗P < 0.001. **(E**–**F)** Cells from (A) were stained with Oil Red O (E), which can stain neutral lipids (Scale bar: 20 μm). Quantification is plotted in (F). Statistical analysis results are shown as mean ± SD; ∗∗∗p < 0.001 by one-way ANOVA, n = 50. **(G**–**I)** Cells from (A) were analyzed by qRT-PCR (G) to determine expression of genes involved in lipolysis and lipogenesis, and by triglyceride (TG) (H) and malonyl-CoA (I) assays. Statistical significance was calculated using the Student's t-test. Results are presented as mean ± SD. ∗∗∗P < 0.001. n = 3**. (J**–**L)** C57BL/6 J mice received intrahepatic injections of siRNAs against ROAM1 (4 nM)(n = 6 per group), and liver tissue close to the injection site was collected on day 4 for analysis (J), including WB (K) and p-AMPK quantification (L). Statistical analysis results are shown as mean ± SD; ∗∗∗p < 0.001 by Student's *t*-test, n = 6. siCtrl, a scramble siRNA. **(M**–**O)** Samples from (J) were analyzed by qRT-PCR (M) to determine expression of genes involved in lipolysis, lipogenesis, and immune response, as well as in TG (N) and malonyl-CoA (O). Statistical significance was calculated using the Student's t-test. mean ± SD; ∗∗P < 0.01, ∗∗∗P < 0.001.

ACC is responsible for catalyzing the first committed step in fatty acid biosynthesis [41]. Phosphorylation of ACC by AMPK inhibits its activity and regulates lipid metabolism [42,43]. We noticed that knocking down ROAM1 in HepG2 and HEK293T cells not only elevated ACC phosphorylation but also sharply reduced lipid droplets (Supplementary Fig. S2A–D), indicating increased fatty acid oxidation and decreased fatty acid synthesis. In both ROAM1 KD lines, the phenotype of increased ACC phosphorylation and decreased lipid droplets was reversed by treatment with the potent, selective AMPK inhibitor BAY-3827 (Supplementary Fig. S2A–F). Similar findings were also obtained using AML12 cells, an immortalized mouse hepatocyte cell line that maintains differentiated functions [44]. Upon ROAM1 depletion, AML12 cells exhibited a marked increase in ACC phosphorylation, which was accompanied by a pronounced loss of lipid droplets (Fig. 2A–F). Again, treatment with BAY-3827 abolished these changes (Fig. 2A–F). In addition, genes encoding the fatty acid synthase (*Fasn*) and lipogenic transcription factor *Srebp1c* [19] were downregulated upon ROAM1 KD, whereas lipolysis genes *Atgl* [45] and *Hsl* [46] were upregulated (Fig. 2G). Of note, the reduction in transcriptional levels of lipogenic genes (approximately 50%) was greater than changes in lipolytic gene expression (approximately 15%), suggesting that AMPK activation induced by ROAM1 knockdown preferentially inhibits lipid synthesis rather than lipolysis. Furthermore, the levels of triglycerides and malonyl-CoA, a substrate for fatty acid synthase [47], also significantly decreased (Fig. 2H and I), in line with reduced lipogenic activity in the KD cells. These results combined argue that ROAM1's inhibition of AMPK activity impacts AMPK-dependent regulation of lipid metabolic pathways.

We also determined the role of ROAM1 in regulating AMPK activity and its function *in vivo* using an established *in vivo* siRNA delivery system to knock down ROAM1 in mice [48]. The liver is central to nutrient metabolism, while skeletal muscle supports metabolic homeostasis [49]. To explore the impact of ROAM1-regulated AMPK activity on systemic metabolism *in vivo*, we adopted a localized delivery strategy using a single intrahepatic injection (Fig. 2J) or two intramuscular injections (Supplementary Fig. S3A) of siRNAs targeting ROAM1 in liver and muscle cells, respectively. We observed significant downregulation of mouse ROAM1 in both the liver and muscle at the indicated post-injection time points compared to control mice (Fig. 2K–L and Supplementary Fig. S3B–C). Consistent with our observations in cellular models, knockdown of ROAM1 in mouse liver and muscle markedly increased AMPK phosphorylation without altering total AMPK protein levels. This AMPK activation was accompanied by elevated ACC phosphorylation (Fig. 2K–L and Supplementary Fig. S3B–C), downregulation of Fasn and Srebp1c, upregulation of Atgl and Hsl (without affecting inflammation regulators), as well as decreased levels of triglycerides and malonyl-CoA (Fig. 2M–O and Supplementary Fig. S3D–F). These changes are consistent with a metabolic shift from fatty acid synthesis toward lipid catabolism. Overall, our results support a model in which ROAM1 inhibits AMPK activity and thereby modulates AMPK-downstream lipid metabolic pathways both *in vitro* and *in vivo*, pointing to the potential of developing lysosome-targeting AMPK activators directed against ROAM1 for reducing fat content.

### 2.3 UDP-GlcNAc is a ROAM1 ligand and novel AMPK activator

**Fig. 3:**
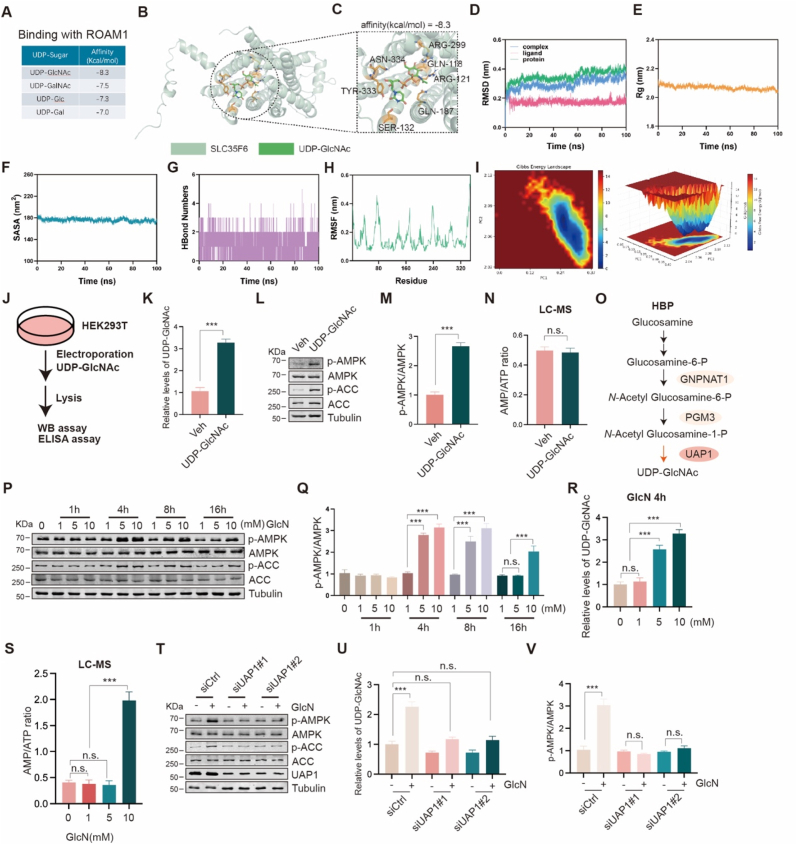
The HBP end product UDP-GlcNAc activates AMPK both *in vitro* and in **vivo. (A)** The table shows the affinity between UDP-sugar and ROAM1. **(B)** Molecular docking reveals the binding mode between ROAM1 and UDP-GlcNAc. **(C)** The predicted binding site of UDP-GlcNAc on ROAM1 is indicated. (D) RMSD, (E) Rg, (F) SASA, (G) H-bonds, (H) RMSF, and (I) free energy landscape of the ROAM1–UDP-GlcNAc complex over simulation time. **(J**–**N)** HEK293T cells electroporated with 200 mM UDP-GlcNAc were cultured for 1 h (J), and harvested for UDP-GlcNAc ELISA assay (K) or lysed in ODG buffers for WB as indicated (L). Quantification of p-AMPK levels is shown in (M) and AMP/ATP ratios as determined by LC-MS in (N). Veh, 1xPBS. Error bars represent mean ± SD, n = 3. Statistical significance was calculated using Student's t-test. ∗∗∗P < 0.001; n.s., not significant. **(O)** Glucosamine (GleN) can enter the hexosamine biosynthesis pathway (HBP) and be converted to UDP-GlcNAc. UAP1 catalyzes the final and rate-influencing step in producing UDP-GlcNAc. **(P**–**Q)** HEK293T cells treated with different concentrations of GlcN for the indicated lengths of time were harvested for WB as indicated (P). Quantification of p-AMPK levels is shown in (Q). Statistical analysis results are shown as mean ± SD; n.s., not significant; ∗∗∗p < 0.001 by one-way ANOVA, n = 3. Vehicle (1xPBS) alone served as negative controls. **(R**–**S)** Cells from (P) that were treated with different concentrations of GlcN for 4 h were collected for UDP-GlcNAc ELISA assay (R) and AMP/ATP ratio determination by LC-MS (S). Statistical analysis results are shown as mean ± SD; n.s., not significant; ∗∗∗p < 0.001 by one-way ANOVA, n = 3. **(T**–**V)** HEK293T cells expressing two different siRNAs against UAP1 (siUAP1) were treated with GlcN (5 mM) for 4 h, and harvested for WB (T) and p-AMPK level quantification (V) as indicated. Results from UDP-GlcNAc ELISA assays are shown in (U). siCtrl, scramble siRNA. Vehicle (1xPBS) alone served as negative controls. Error bars represent mean ± SD, n = 3. Statistical significance was calculated using the Student's t-test. ∗∗∗P < 0.001, n.s., not significant.

ROAM1 is a member of the SLC35 family, which are NSTs with a preference for UDP-linked sugars such as UDP-GlcNAc and UDP-GalNAc. These nucleotide sugars are direct precursors for the synthesis of glycosylated molecules (e.g., polysaccharides, glycoproteins, and glycolipids) [[50], [51], [52], [53]]. Notably, many glycoconjugates synthesized from UDP-sugars are essential in the pathobiology of diseases like cancer and type 2 diabetes, conditions where glucose metabolism is dysfunctional [53,54]. Given that UDP-sugars may be natural ligands of ROAM1 capable of activating AMPK and potentially treating MASLD, we performed molecular docking to predict the affinity between UDP-sugars and ROAM1. As shown in Fig. 3A, among several common SLC35 family ligands, UDP-GlcNAc exhibited the strongest affinity for ROAM1. This binding occurred within the transmembrane region of ROAM1 (Fig. 3B) and was dependent on key amino acid residues, including GLN-118, ARG-121, SER-132, GLN-187, ARG-299, TYR-333, and ASN-334 (Fig. 3C). To further validate the binding stability, we performed molecular dynamics (MD) simulations of the UDP-GlcNAc–ROAM1 complex [55]. As shown in Fig. 3D, the complex reached equilibrium after 60 ns with RMSD fluctuations around 0.35 nm, indicating good conformational stability. The radius of gyration (Rg) remained stable throughout the simulation (Fig. 3E), and no significant changes in solvent-accessible surface area (SASA) were observed (Fig. 3F), collectively suggesting a compact and stable binding interface. Hydrogen bond analysis revealed an average of approximately 3 hydrogen bonds between UDP-GlcNAc and ROAM1 during the simulation (Fig. 3G), supporting favorable interactions. RMSF analysis showed that most residues exhibited values below 0.45 nm (Fig. 3H), indicating low flexibility of the binding region. Free energy landscape analysis identified a low-energy conformation state (Fig. 3I), further confirming the stability of the complex. Taken together, these MD simulation results provide strong dynamic evidence supporting the binding mode predicted by molecular docking and demonstrate that UDP-GlcNAc forms a stable complex with ROAM1. However, since UDP-sugars are unable to penetrate the cell membrane, we initially evaluated the impact of these UDP-sugars on AMPK activity under *in vitro* conditions. After cell lysates were incubated with several common UDP-sugars (Supplementary Fig. S3G), heightened AMPK phosphorylation could be readily detected in all the samples with very high UDP-sugar concentrations (Supplementary Fig. S3H–O). Interestingly, only UDP-GlcNAc promoted AMPK phosphorylation at low millimolar concentration (Supplementary Fig. S3L–M), a level closer to its physiological concentration in mammalian cells [56]. Phosphorylation of AMPK appeared to reach plateau at 2 mM UDP-GlcNAc, showing no discernible increase with additional UDP-GlcNAc (Supplementary Fig. S4A–B). These results suggest that UDP-GlcNAc may be the primary UDP-sugar involved in AMPK regulation. As expected, directly adding UDP-GlcNAc to cultured cells failed to elicit any detectable changes in AMPK activity (Supplementary Fig. S4C–D). When we employed electroporation, intracellular UDP-GlcNAc levels was boosted by approximately threefold (Fig. 3J–K). This increase was accompanied by a marked rise in the phosphorylation level of both AMPK and ACC (Fig. 3L–M), while the AMP/ATP ratio remained unchanged (Fig. 3N). A time-course analysis after UDP-GlcNAc electroporation showed that AMPK phosphorylation peaked around 60 min and returned to basal levels after 4 h (Supplementary Fig. S4E–F).

To better investigate the effect of UDP-GlcNAc on AMPK, we switched to glucosamine (GlcN), which is readily taken up by cells and can enter the hexosamine biosynthesis pathway (HBP) downstream of GFAT to robustly elevate intracellular UDP-GlcNAc (Fig. 3O) [[57], [58], [59]]. Indeed, culturing cells with GlcN (5 mM or 10 mM) for 4 h led to the expected increase in AMPK and ACC phosphorylation (Fig. 3P–Q). The 5 mM dose, which effectively elevated UDP-GlcNAc levels without altering the AMP/ATP ratio (Fig. 3R–S), was chosen for subsequent experiments. To confirm that GlcN activates AMPK specifically through UDP-GlcNAc, we added GlcN to cells expressing siRNAs against UAP1, the final enzyme in the HBP (Fig. 3O) [60]. Treatment with two different UAP1-targeting siRNAs successfully suppressed UAP1 protein expression (Fig. 3T), and reduced intracellular UDP-GlcNAc levels (Fig. 3U). Importantly, UAP1 KD abolished the increase in AMPK and ACC phosphorylation induced by GlcN treatment (Fig. 3T and V), supporting the notion that GlcN activates AMPK via the HBP end product UDP-GlcNAc. Although GlcN is the primary source for generating UDP-GlcNAc through the HBP, mannose can also enter the HBP and serve as a precursor for UDP-GlcNAc [[61], [62], [63]]. Consistent with the results using GlcN, culturing cells with mannose led to detectable increases in the phosphorylation of AMPK and ACC by 4 h and marked increases by 8 h (Supplementary Fig. S4G–H). The above results combined demonstrate that exposure to elevated UDP-GlcNAc leads to activation of AMPK above basal levels in cells.

Given that UDP-GlcNAc is the donor substate for O-GlcNAcylation [64,65], which can also modulate the activity of AMPK, we examined the time course of global O-GlcNAcylation changes following GlcN addition to assess the role of this modification in UDP-GlcNAc-mediated regulation of AMPK. Treatment with UDP-GlcNAc for 4 h did not result in significant changes in global O-GlcNAcylation levels (Supplementary Fig. S4I–J), whereas AMPK activation could be detected as early as 1 h after treatment (Supplementary Fig. 4E–F). Importantly, even in the presence of the O-GlcNAc transferase (OGT) inhibitor OSMI-1, which blocks O-GlcNAcylation, UDP-GlcNAc was still able to enhance AMPK and ACC phosphorylation (Supplementary Fig. S4K–L). These results indicate that UDP-GlcNAc activates AMPK independently of O-GlcNAcylation. Exposing cells to high concentrations of small solutes (e.g., sorbitol) or permeant sugars (e.g., glucose and glucosamine) can raise extracellular osmolarity and induce hyperosmotic stress [66,67]. To address whether AMPK activation observed in the above experiments was due to osmotic stress, we assessed the phosphorylation level of the Yes-associated protein (YAP), a sensitive osmotic stress sensor [68,69]. As expected, high concentration of sorbitol [70,71] induced an increase in the phosphorylation of YAP and AMPK in cells (Supplementary Fig. S4M–N). In contrast, treatment with UDP-GlcNAc, GlcN, or mannose at the indicated doses did not impact YAP phosphorylation. These data do not support a role of osmotic stress in AMPK activation in cells treated with low millimolar concentrations of UDP-GlcNAc, GlcN, or mannose. Collectively, our results point to UDP-GlcNAc as a specific metabolite activator of AMPK, operating through pathways separate from O-GlcNAcylation and osmotic stress.

### 2.4 UDP-GlcNAc activates AMPK via ROAM1

Mechanistically, we next examined whether UDP-GlcNAc acts through ROAM1. As shown in Supplementary Fig. S5A–B, lysates from ROAM1 KD cells were incubated with different concentrations of UDP-GlcNAc. Even at very high concentrations, UDP-GlcNAc failed to induce AMPK phosphorylation. Likewise, no enhanced phosphorylation of AMPK or ACC was observed upon UDP-GlcNAc electroporation or addition of GlcN or mannose into ROAM1 KD cells (Supplementary Fig. S5C–H), indicating that ROAM1 is required for UDP-GlcNAc-induced AMPK activation. Furthermore, electroporation of UDP-GlcNAc into doxycycline-induced ROAM1 KO cells also failed to elevate the phosphorylation of AMPK or ACC (Supplementary Fig. S5I–J). This failure contrasts with the cells’ response to several established AMPK activators, including AICAR, H_2_O_2_, and glucose starvation, which all augmented AMPK and ACC phosphorylation regardless of ROAM1 status (Supplementary Fig. S5I–J). These findings strongly support the notion that UDP-GlcNAc and ROAM1 act in the same pathway to regulate AMPK through a mechanism distinct from conventional activation routes.

ROAM1 harbors a conserved 10-transmembrane domain (TM domain) that is found in all SLC35 family members and known for binding to cargo such as UDP-GlcNAc [72,73]. We next generated a TM domain deletion mutant (Supplementary Fig. S5K), and expressed it in ROAM1 KO cells (Supplementary Fig. S6A). Consistent with our data above, electroporating UDP-GlcNAc into ROAM1 KO cells expressing full-length ROAM1 led to increased phosphorylation of AMPK and ACC (Supplementary Fig. S6B–C). However, such response was abolished in KO cells expressing the TM domain deletion mutant. ROAM1's C-terminal tail (C-tail) contains a putative lysosomal targeting motif (Supplementary Fig. S5K). Given that the β subunits are key to enabling AMPK heterotrimer localization to the lysosomal membrane [38], we hypothesized that the C-tail may help mediate the interaction between ROAM1 and AMPKβ. Indeed, deletion of the C-tail disrupted their association (Supplementary Fig. S6D). When electroplated with UDP-GlcNAc, ROAM1 KO cells expressing the C-tail truncation mutant also failed to upregulate AMPK/ACC phosphorylation (Supplementary Fig. S6A, E-F). We further validated this by mutating the lysosomal sorting motif (EXXXL to AXXXA) in ROAM1. This mutant lost lysosomal localization (Supplementary Fig. S6G) and largely abrogated UDP-GlcNAc-induced AMPK activation (Supplementary Fig. S6H–I), confirming that lysosomal localization is essential for ROAM1-mediated AMPK activation. These findings suggest a model in which UDP-GlcNAc is brought closer to AMPKβ through the combined action of the cargo-binding TM domain and AMPKβ-binding C-tail of ROAM1. Thus, ROAM1 likely functions as a scaffold protein that facilitates the formation of a functional activation complex essential for AMPK regulation.

**Fig. 4:**
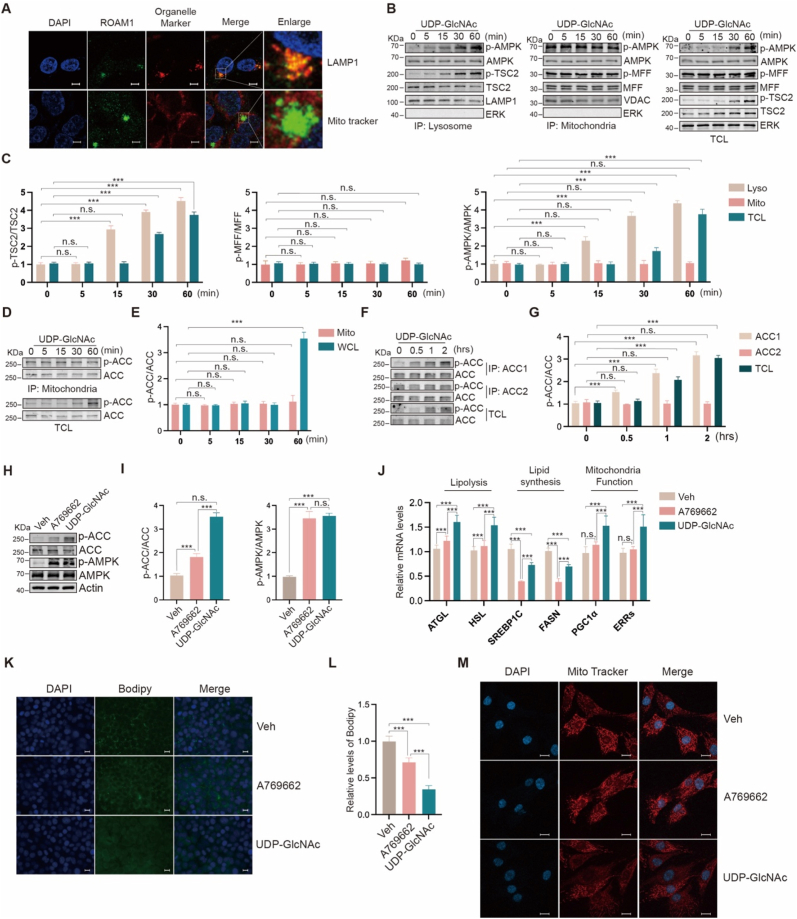
ROAM1 is required for AMPK activation by UDP-G**lcNAc. (A)** HEK293T cells were co-stained with an anti-ROAM1 antibody (green) and either an anti-LAMP1 antibody (red) to mark lysosomes or MitoTracker (red) to label mitochondria. DAPI was used to stain nuclei (blue). Scale bar: 5 μm. Boxed regions are enlarged and shown on the right. **(B**–**E)** AML12 cells expressing HA-tagged OMP25 or Flag-tagged TMEM192 were electroporated with UDP-GlcNAc (200 mM) and cultured for the indicated times. Cells were then harvested for lysosome enrichment via anti-Flag IP or for mitochondrial enrichment via anti-HA IP, followed by WB as shown in (B) and (D). (C, E) Quantification of p-AMPK, p-TSC2, or p-MFF levels in lysosomal and mitochondrial fractions, as well as p-ACC levels in mitochondrial fractions and TCL, are presented as mean ± SD (n = 3). n.s., not significant; ∗∗∗p < 0.001 by one-way ANOVA. Vehicle (1× PBS) alone served as negative control. TCL, Total-cell lysates. **(F, G)** AML12 cells were lysed for endogenous ACC1 and ACC2 IP, followed by Western blotting (WB) as shown in (F). Quantification of p-ACC levels are presented as mean ± SD (n = 3). n.s., not significant; ∗∗∗p < 0.001 by one-way ANOVA. **(H**–**M)** AML12 cells were treated with A769662 (400 μM) and electroporated with UDP-GlcNAc (200 mM) for 2 days. Cells were then collected for WB to detect p-AMPK and p-ACC (H), qRT-PCR analysis of the indicated lipolysis and lipogenesis genes (J), lipid droplet staining with BODIPY 493/503 (K), and mitochondrial staining with MitoTracker (M). Quantification of p-AMPK and p-ACC levels is presented in (I). Fluorescence signals of (K) were quantified and plotted in (L). Vehicle (1× PBS) served as control. Error bars represent mean ± SD (n = 3). Statistical significance was calculated using one-way ANOVA. ∗∗∗P < 0.001. Scale bar: 10 μm.

Next, we further validated the specificity of ROAM1 among SLC35 family members for AMPK lysosomal activation. Although the TM domain is conserved among SLC35 family proteins [73], AMPK activation was not observed in cells depleted of SLC35A3 or SLC35D1 (Supplementary Fig. S7A–H), two SLC35 family proteins known to transport UDP-GlcNAc [74]. This is likely due to their lack of lysosomal localization motifs. Sequence analysis indicates that ROAM1 is the least conserved member of the SLC35F subfamily (Supplementary Fig. S8A–B). Unlike other SLC35F proteins such as SLC35F2, SLC35F3, and SLC35F5, whose immunostaining signals were observed on the plasma membrane and in the cytoplasm, overlapping with the Golgi marker, ROAM1 signals were mostly absent from the plasma membrane and only partly co-localized with the Golgi marker (Supplementary Fig. S9A). Using an anti-ROAM1 antibody validated with inducible ROAM1 KO cells (Supplementary Fig. S9B), we found endogenous ROAM1 to co-localize with the lysosomal marker LAMP1 but not with mitochondrial markers (Fig. 4A and Supplementary Fig. S9C). Furthermore, in AML12 cells ectopically expressing Flag-tagged TMEM192, a protein primarily localized to lysosomal and late endosomal membranes and widely used for lysosome purification [75,76], anti-Flag IP could bring down both ROAM1 and the lysosomal marker LAMP1 but not the cytoplasmic protein ERK (Supplementary Fig. S9D). Indeed, ROAM1, but not SLC35F2/3/5, could co-IP with AMPK β1 and β2 (Supplementary Fig. S9E–F). This association was greatly reduced when the Gly2 residue in AMPKβ was mutated, which prevents myristoylation and membrane anchoring (Supplementary Fig. S9G), indicating that ROAM1-AMPKβ interaction depends on the membrane localization of AMPKβ. Together, these results indicate that the lysosomal localization of ROAM1, unique among SLC35 family members, is essential for UDP-GlcNAc-induced AMPK activation.

To elucidate the mechanism by which ROAM1 negatively regulates AMPK, we investigated whether ROAM1 recruits a protein phosphatase to promote AMPKα dephosphorylation. PP2C is a well-established phosphatase for AMPK dephosphorylation [77]. In an *in vitro* dephosphorylation assay, purified phosphorylated AMPK was incubated with PP2C in the presence or absence of ROAM1. In the absence of ROAM1, a significant reduction in AMPK phosphorylation was observed only after approximately 60 min; however, in the presence of ROAM1, marked dephosphorylation was detected as early as 15 min (Supplementary Fig. S10A–C), indicating that ROAM1 directly enhances PP2C-mediated AMPK dephosphorylation. Co-immunoprecipitation experiments further confirmed a specific interaction between ROAM1 and PP2C. Functionally, overexpression of ROAM1 together with PP2C led to a significantly greater reduction in AMPKα Thr172 phosphorylation compared to the GFP control, and this effect was effectively reversed by UDP-GlcNAc treatment, which weakened the ROAM1-PP2C interaction (Supplementary Fig. S10D). Collectively, these results support a model in which ROAM1 acts as a scaffold to recruit PP2C to the AMPK complex, enhancing AMPKα dephosphorylation at Thr172. UDP-GlcNAc, as the natural ligand of ROAM1, relieves this inhibition by disrupting the ROAM1-PP2C interaction, thereby activating AMPK. This "dephosphorylation–ligand dissociation–activation" module positions ROAM1 as a molecular switch within the lysosomal AMPK signaling network.

### 2.5 UDP-GlcNAc activates lysosomal AMPK to primarily phosphorylate metabolic substrates and regulate lipid metabolism

Given that UDP-GlcNAc's regulation of AMPK appears to depend on the lysosomal transmembrane protein ROAM1, we next asked whether UDP-GlcNAc preferentially activates lysosomal AMPK in a substrate-selective manner. To better understand the substrate preference of the UDP-GlcNAc-ROAM1-AMPK axis in the liver, we electroporated UDP-GlcNAc into normal mouse hepatocyte AML12 cells ectopically expressing Flag-TMEM192 or HA-OMP25 [78]. Subsequently, we isolated lysosomal fractions via anti-Flag immunoprecipitation (Supplementary Fig. S9D). Within 15 min of UDP-GlcNAc treatment, we detected increased phosphorylation of AMPK and its lysosomal substrate RAPTOR and TSC2 (Fig. 4B–C and Supplementary Fig. S10E–I). In contrast, in mitochondrial fractions enriched by anti-HA IP (Supplementary Fig. S9H), we could not detect activation of AMPK or its mitochondrial substrate MFF (which is involved in mitochondrial fission) (Fig. 4B–C). In the cytoplasmic fraction, we also observed significant phosphorylation of the lysosomal substrate TSC2, but no increase in phosphorylation of the mitochondrial-associated substrate MFF (Fig. 4B–C). The cytosolic AMPK substrate ULK1 displayed only weak phosphorylation at the lysosome and remained unphosphorylated in the cytosolic fraction (Supplementary Fig. S10E–I). Moreover, we found prominent ACC phosphorylation in the cytoplasm, whereas no such phosphorylation was detected on mitochondria (Fig. 4D–E), demonstrating compartmentalized regulation of ACC phosphorylation by AMPK. Of the two ACC isoforms, cytoplasmic ACC1 is primarily involved in lipid synthesis whereas mitochondria-associated ACC2 is mostly involved in regulating lipid oxidation. Because the ACC antibody recognizes both ACC1 and ACC2 [18], we immunoprecipitated ACC1 and ACC2 and examined their activation in AML12. Upon UDP-GlcNAc addition, markedly increased activation of ACC1, but not ACC2, was observed (Fig. 4F–G). This finding is consistent with the lack of activation of the mitochondrial AMPK substrate MFF. These findings also align well with our earlier observations in ROAM1 KD cells (Fig. 2G), where genes involved in fatty acid synthesis were strongly downregulated while lipolytic genes were only modestly upregulated. Taken together, these results indicate that UDP-GlcNAc-ROAM1-mediated lysosomal AMPK activation preferentially phosphorylates lysosomal and metabolic substrates, especially those driving lipid synthesis, thereby reducing fat content.

We further compared UDP-GlcNAc with the classic AMPK activator A769662. After these activators were introduced into AML12 cells for 24 h, comparable levels of AMPK phosphorylation were detected, although UDP-GlcNAc appeared to induce ACC phosphorylation to a greater degree (Fig. 4H–I). Moreover, UDP-GlcNAc preferentially downregulated genes involved in lipid synthesis (e.g., SREBP1C and FASN) and only mildly affected genes related to lipolysis (e.g., ATGL and HSL), while A769662 elicited more balanced transcriptional changes in AMPK downstream genes. (Fig. 4J). At the metabolic level, UDP-GlcNAc markedly reduced lipid droplet content but had little effect on mitochondrial fission (Fig. 4K–M). In contrast, A769662 had moderate impact on both lipid droplet reduction and mitochondrial fission (Fig. 4K–M). These results indicate that UDP-GlcNAc activates AMPK in a manner more selective than A769662, preferentially modulating metabolism, particularly lipid synthesis-related proteins. Thus, UDP-GlcNAc may be better suited for activating AMPK to downregulate lipid synthesis, positioning it as a more ideal therapeutic for MASLD.

### 2.6 The UDP-GlcNAc-derived nanosheet MgUGN enables *in vivo* investigation of UDP-GlcNAc

**Fig. 5:**
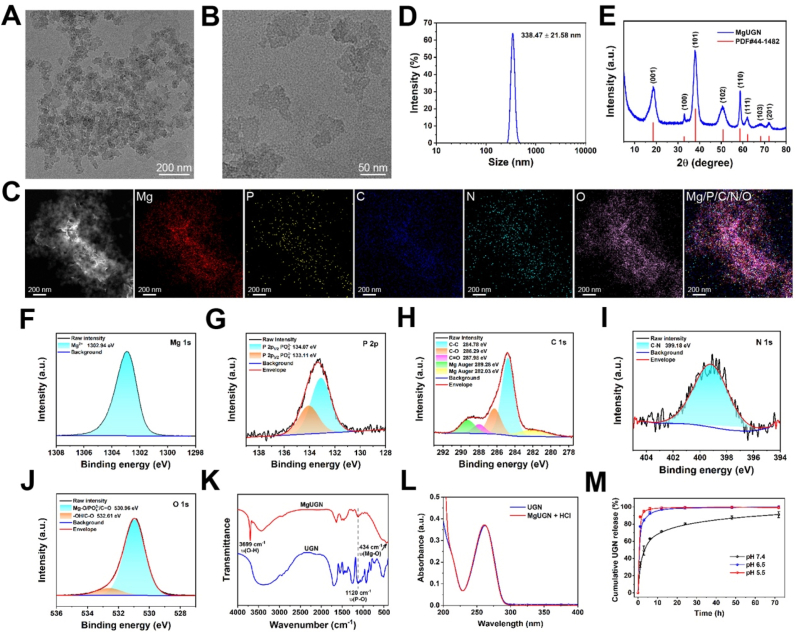
UDP-GlcNAc assembles into magnesium-coordinated nanosheets (MgUGN) with defined structural features and effective rele**ase. (A**–**B)** Representative TEM images of MgUGN nanosheets at different magnifications. Scale bars: 200 nm (A) and 50 nm (B). **(C)** Elemental mapping of MgUGN nanosheets for the elements Mg, P, C, N, and O. **(D)** Hydrodynamic size distribution of MgUGN nanosheets as measured by dynamic light scattering (DLS). Results are presented as mean ± SD, n = 3. **(E)** XRD pattern of MgUGN nanosheets, with reflections (Blue) matching the Mg(OH)_2_ crystalline phase (PDF# 44-1482) (Red). **(F**–**J)** High-resolution XPS spectra of Mg 1s (F), P 2p (G), C 1s (H), N 1s (I), and O 1s (J) in the MgUGN nanosheets. **(K)** FTIR spectra of pure UDP-GlcNAc and MgUGN nanosheets. Characteristic peaks for Mg–O and P–O bonds were observed at 434 cm^−1^ and 1120 cm^−1^, respectively, in the MgUGN spectrum. **(L)** UV-Vis absorption spectra of a pure UDP-GlcNAc aqueous solution and a solution of MgUGN nanosheets dissolved in HCl. **(M)** The MgUGN nanosheets were dispersed in 1x PBS with the indicated pH values. The cumulative release profile of UDP-GlcNAc was plotted based on UV-Vis absorbance at 262 nm (mean ± SD, n = 3).

Although UDP-GlcNAc can selectively activate lysosomal AMPK to preferentially reduce fat content, its poor permeability and transient activation (<4 h) severely limit *in vivo* studies. Similar challenges of poor solubility, chemical instability, and low bioavailability are often common to natural compounds. Tremendous progress has been made in delivery systems to help overcome such obstacles, including lipid-based carriers, polymer nanosheets, inclusion complexes, micelles, and conjugate-based platforms [79]. For instance, EGCG has been formulated into nanosheets to enhance its AMPK activation capacity [80], and nanoinhibitors targeting lysosomal V-ATPase have been engineered to enhance lysosomal AMPK signaling [81]. To improve UDP-GlcNAc cellular uptake, we performed a one-pot precipitation reaction that mixed UDP-GlcNAc with magnesium ions under mildly alkaline conditions, which led to the formation of small, nanosheet-like structures (Fig. 5A–B). We named these magnesium ion-coordinated nanosheets MgUGN and characterized their structural properties. Elemental mapping revealed a homogeneous distribution of Mg, P, C, N, and O throughout the nanosheets (Fig. 5C). The presence of these elements was further verified by X-ray photoelectron spectroscopy (XPS) (Fig. 5F–J and Supplementary Fig. S10J). The phosphorus (P) element is intrinsic to the UDP-GlcNAc molecule, and its presence confirms that UDP-GlcNAc successfully coordinated with magnesium ions to form stable nanosheets. Dynamic light scattering (DLS) measurements showed that the nanosheets had a hydrodynamic diameter of 338.47 ± 21.58 nm (Fig. 5D), and zeta potential analysis indicated a negatively charged surface (Supplementary Fig. S10K). X-ray diffraction (XRD) confirmed that the nanosheets adopted the crystalline phase of Mg(OH)_2_ (Fig. 5E), whose formation was facilitated by the mildly alkaline reaction conditions. The absence of the amorphous UDP-GlcNAc diffraction peak in MgUGN, along with homogeneous phosphorus distribution and characteristic Mg–O/P–O bonds, supports the formation of a coordination structure rather than a physical mixture of Mg(OH)_2_ and UDP-GlcNAc (Supplementary Fig. S10L). Fourier transform infrared (FTIR) spectroscopy revealed characteristic absorption bands at 434 cm^−1^ and 1120 cm^−1^, corresponding to Mg–O and P–O bonds, respectively (Fig. 5K), providing additional confirmation of successful coordination and the formation of stable MgUGN nanosheets.

Free UDP-GlcNAc in aqueous solution exhibited a prominent UV-Vis peak at 262 nm (Supplementary Fig. S10M–N), and absorbance at this wavelength scaled linearly with concentration, providing a reliable readout for quantifying UDP-GlcNAc amount in the nanosheets. MgUGN dissolved readily in acid, and upon dilution in hydrochloric acid, its UV-Vis spectrum closely resembled that of pure UDP-GlcNAc, retaining the same characteristic peak at 262 nm (Fig. 5L). We calculated the amount of UDP-GlcNAc in MgUGN to be 12.87 wt% based on UV-Vis measurements. These results suggest that UDP-GlcNAc successfully assembles into nanosheets without altering its molecular structure. To evaluate content release profiles, MgUGN nanosheets were dispersed in 1xPBS at different pH and UDP-GlcNAc release rates were calculated based on UV-Vis absorption at 262 nm. Higher acidity and longer incubation times accelerated UDP-GlcNAc release, reaching 97.85 ± 1.59% after 6 h at pH 5.5 (Fig. 5M). These data show that MgUGN nanosheets remain stable and release UDP-GlcNAc slowly under neutral conditions, while acidic conditions trigger rapid release, a desirable property for targeting acidic subcellular organelles like lysosomes.

### 2.7 MgUGN is a potent AMPK activator and enables sustained AMPK signaling

**Fig. 6:**
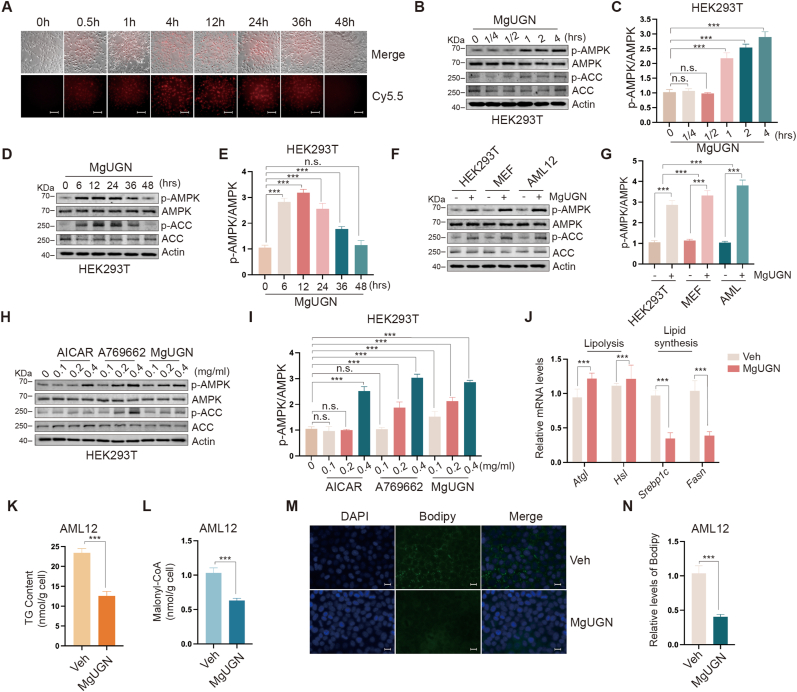
MgUGN potently activates **AMPK. (A)** HEK293T cells incubated with Cy5.5-labeled MgUGN (100 μg/mL) were imaged live at different time points, and fluorescence signals (red) were overlaid with the corresponding bright-field images. Scale bar: 25 μm. **(B**–**E)** HEK293T cells incubated with MgUGN (100 μg/mL) were harvested at different time points for WB as indicated in (B) and (D), with p-AMPK quantification in (C) and (E). Vehicle alone (1xPBS) served as negative controls. Error bars represent mean ± SD, n = 3. Statistical significance was calculated by one-way ANOVA. ∗∗∗P < 0.001. n.s., not significant. **(F**–**G)** MgUGN was added to HEK293T cells (100 μg/ml), mouse embryonic fibroblasts (MEFs) (50 μg/ml), and AML12 cells (50 μg/ml) for 1 h before WB (F), with p-AMPK quantification in (G) as mean ± SD; ∗∗∗p < 0.001 by one-way ANOVA, n = 3. Vehicle (1xPBS) alone served as negative controls. **(H**–**I)** HEK293T cells treated with different concentrations of AICAR, A769662, or MgUGN for 1 h were analyzed by WB (H), with p-AMPK quantification in (I). Vehicle alone (1xPBS) served as negative controls. Error bars represent mean ± SD, n = 3. Statistical significance was calculated using the Student's t-test. ∗∗∗P < 0.001. n.s., not significant. **(J**–**N)** AML12 cells cultured in MgUGN (50 μg/mL) for 24 h were collected for qRT-PCR analysis of the indicated lipolysis and lipogenesis genes (J), content analysis of triglycerides (TG)(M) and malonyl-CoA (N), and lipid droplet staining with BODIPY 493/503 (green) and Hoechst 33342 (blue)(M). Fluorescence quantification from (M) is plotted in (N). Veh, 1xPBS. Error bars represent mean ± SD, n = 3. Statistical significance was calculated using the Student's t-test. ∗∗∗P < 0.001. Scale bar: 10 μm.

To better assess the cellular uptake of MgUGN, we labeled MgUGN with Cy5.5 and incubated HEK293T cells with Cy5.5-MgUGN to monitor fluorescence over time. Intracellular fluorescence was detectable within 30 min, peaked at 4 h, declined after 36 h, and became undetectable by 48 h (Fig. 6A). MgUGN's efficient cellular internalization and intracellular stability are supported by sustained AMPK activation in these cells. Similar to UDP-GlcNAc, MgUGN elicited increased AMPK and ACC phosphorylation within 1 h of treatment (Fig. 6B–C). Notably, AMPK/ACC phosphorylation remained elevated for 24 h (∼3-fold above baseline), and was still 1.5-fold higher at 36 h, returning to baseline after 48 h (Fig. 6D–E). These findings demonstrate that MgUGN substantially outperforms UDP-GlcNAc at inducing sustained AMPK activation. Dose-response analysis revealed significant increases in AMPK/ACC phosphorylation in HEK293T cells starting at 100 μg/mL of MgUGN, with only modest additional elevation up to 800 μg/ml (Supplementary Fig. S11A–B). This effective dose is considerably lower than the dose needed for electroporating UDP-GlcNAc (607 μg/ml, 1 mM). MgUGN was also able to activate AMPK in mouse embryonic fibroblasts (MEFs) and immortalized hepatocytes (AML12), which exhibited robust phosphorylation increases at an even lower dose (50 μg/mL) (Fig. 6F–G). It is known that A-769662 achieves more potent AMPK activation than AICAR without affecting other AMP-sensitive enzymes such as fructose-1,6-bisphosphatase or glycogen phosphorylase [82]. As expected, cells treated with A769662 exhibited strong AMPK activation at lower concentrations (200 μg/ml) than those with AICAR (400 μg/ml), yet both compounds required higher doses than MgUGN (Fig. 6H–I), underscoring the markedly higher potency of MgUGN in activating AMPK in cells.

The observed MgUGN-stimulated AMPK activation should be specific to the assembled nanosheet complex, as only cells incubated with MgUGN, but not MgCl_2_ or UDP-GlcNAc, showed increased AMPK/ACC phosphorylation (Supplementary Fig. S11C–D). Since UDP-GlcNAc self assembles into MgUGN, they are expected to function in the same manner to activate AMPK. To test this, we again turned to cells depleted of UAP1, which impairs the conversion of GlcN to UDP-GlcNAc (Fig. 3O). As predicted, UAP1 KD cells treated with MgUGN, but not GlcN, exhibited increased UDP-GlcNAc levels as well as elevated AMPK/ACC phosphorylation (Supplementary Fig. S11E–G). Furthermore, we failed to detect induced AMPK activation in ROAM1 KO cells treated with MgUGN (Supplementary Fig. S11H–I), reminiscent of the responses of ROAM1 KO cells to UDP-GlcNAc (Supplementary Fig. S5I–J). These results support the notion that UDP-GlcNAc is the bioactive agent in MgUGN and requires ROAM1 for AMPK activation. Importantly, the robust AMPK activation stimulated by MgUGN treatment was accompanied by changes in metabolic pathways regulated by AMPK. In MgUGN-treated AML12 cells, in addition to increased ACC phosphorylation, the expression of lipogenic genes (*Fasn* and *Srebp1c*) was significantly downregulated, while the expression of lipolysis genes (*Atgl* and *Hsl*) was moderately upregulated (Fig. 6J). In line with these changes, the levels of triglycerides and malonyl-CoA also decreased (Fig. 6K–L), suggesting a shift into a lipid-catabolic state. Moreover, a significant decrease in BODIPY (Fig. 6M–N) and Oil Red O (Supplementary Fig. S11J–K) staining was apparent in these cells, indicating reduced lipid droplet content. This drop in lipid content was reversed when the AMPK inhibitor BAY-3827 was added to MgUGN-treated cells (Supplementary Fig. S11J–M). The above observations mirror those obtained with ROAM1 KD cells (Fig. 2A–F and Supplementary Fig. S2A–D), and demonstrate that MgUGN's activation of AMPK signaling led to suppressed lipogenesis and enhanced lipolysis, culminating in reduced cellular lipid content. Moreover, similar to UDP-GlcNAc, MgUGN activity depends on the ROAM1-AMPKβ axis.

Preclinical studies show that combining pharmacological activators and activating signals, such as metformin with exercise or salicylate, and AICAR or A-769662 with stress, can markedly amplify AMPK activation [83,84]. We hypothesized that MgUGN may also enhance the effects of well-known AMPK activators. In MgUGN-treated AML12 cells also exposed to A769662, glucose starvation, or AICAR, AMPK and ACC phosphorylation was higher than in cells treated with individual activators alone (Supplementary Fig. S12A–F). Given that ROAM1 mediates UDP-GlcNAc/MgUGN activity and ROAM1 negatively regulates AMPK, we also investigated the effects of AMPK activating agents and signals on ROAM1 KD cells. A similar potentiation was observed in ROAM1 KD cells receiving the same treatments (Supplementary Fig. S12G–L). These results indicate that MgUGN treatment and ROAM1 inhibition can further enhance AMPK activation when combined with classical AMPK activators, highlighting their potential for combination strategies targeting aging-related and metabolic diseases.

### 2.8 MgUGN provides metabolic benefits and hepatoprotection across different mouse models

It is well established that lipid metabolism is the principal metabolic target of AMPK, with AMPK potently inhibiting lipid and cholesterol synthesis and promoting lipid catabolism to restore energetic balance over the long term [17,85,86]. Given our data on MgUGN, particularly its ability to shift hepatocytes toward a lipid-catabolic state, we reasoned that it could prove effective *in vivo* as well, including in liver disease mouse models. We first administered MgUGN intravenously to healthy mice to evaluate its biosafety. The hematological profiles and hepatic (ALT and AST) and renal (BUN and CREA) function markers in these mice were all comparable to controls (Supplementary Fig. S13A–P), indicating no systemic toxicity. Histopathological examination by H&E staining also found no observable tissue damage or pathological abnormalities in major organs (Supplementary Fig. S14A–F). Taken together, these results demonstrate that MgUGN exhibits excellent biocompatibility and minimal *in vivo* toxicity. Next, we assessed the biodistribution of MgUGN by intravenously injecting Cy5.5-labeled MgUGN into healthy mice for fluorescence imaging. *Ex vivo* analysis of major organs showed the liver displaying the strongest fluorescence, substantially higher than the heart, spleen, lung, or kidneys (Supplementary Fig. S15A–B), indicating efficient hepatic targeting by the MgUGN nanosheets. The liver signal declined gradually over time, consistent with reduced hepatic retention. Notably, AMPK phosphorylation levels remained significantly enhanced in the liver 48 h after MgUGN administration (Supplementary Fig. S15C–D). Together, these data show that MgUGN achieves strong and selective liver accumulation, supporting its potential for liver-directed therapy.

**Fig. 7:**
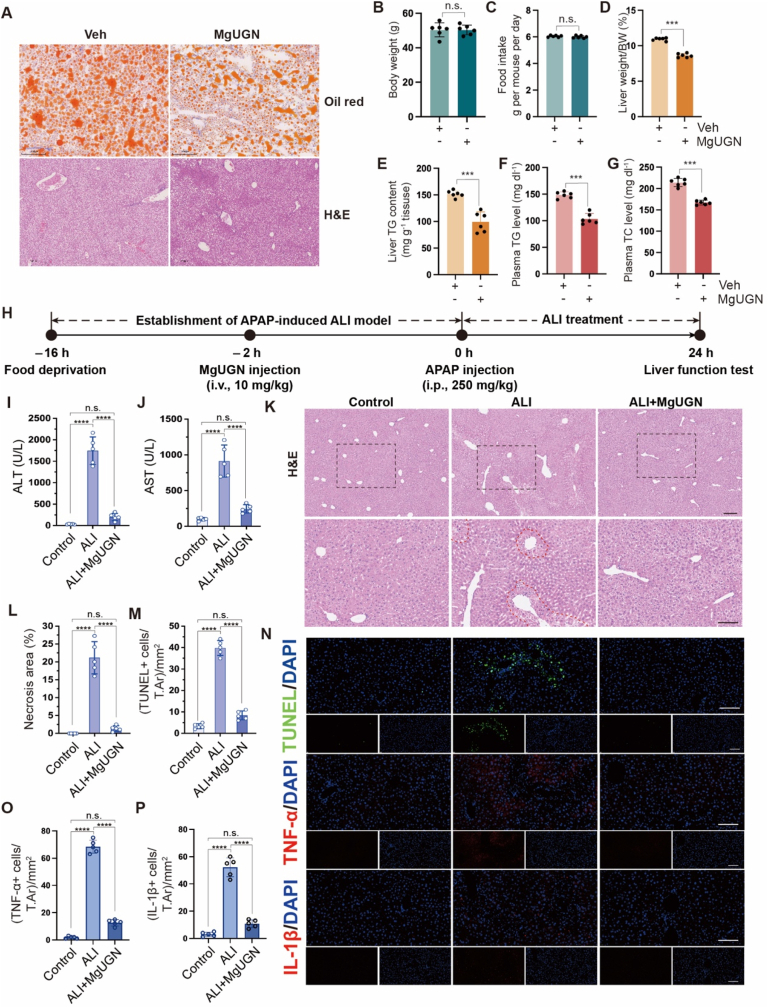
MgUGN improves outcomes across metabolic and acute liver injury mod**els. (A**–**G)***ob/ob* mice received MgUGN (10 mg/kg) through tail-vein injection every 72 h for 14 days. Veh, 1xPBS. Liver tissues were collected for staining (A) with H&E (Scale bar, 500 μm) and Oil Red O (Scale bar, 200 μm). Body weight (B), mean food intake over three days (C), liver/body weight ratio (D), triglyceride (TG) levels in liver (E) and plasma (F), and total cholesterol (TC) in plasma (G) are also shown as mean ± SD; n = 6 mice per group. Statistical significance was calculated using the Student's t-test. ∗∗∗P < 0.001. n.s., not significant. **(H**–**N)** For the acute liver injury (ALI) model (H), C57/B6 mice received MgUGN (10 mg/kg) intravenously 2 h before intraperitoneal APAP administration (250 mg/kg). At 24 h after APAP exposure, blood was collected to assess serum alanine aminotransferase (ALT) (I) and aspartate aminotransferase (AST) (J) levels. Representative H&E staining (K) and quantification (L), as well as TUNEL, TNF-α and IL-1βstaining, quantification (T.Ar)(M, O, P) and representative images (N) of liver tissue sections are also shown. Scale bars: 100 μm. Statistical analysis results are shown as mean ± SD; ∗∗∗∗p < 0.0001 by one-way ANOVA, n = 5 mice per group. n.s., not significant. Nucleus was stained with DAPI.

We then turned to two well-established models of non-alcoholic fatty liver disease (NAFLD) to investigate the therapeutic potential of MgUGN. Wild-type mice on a high-fat-diet (HFD) typically develop steatosis after only a few weeks, and they are widely used models for studying diet-induced metabolic stress [87]. C57BL/6 J mice on 12 weeks of HFD were administered MgUGN via tail vein injection every 72 h for 14 days. Consistent with our findings that MgUGN can enhance lipid catabolism, MgUGN treatment was able to reverse hepatic steatosis and hyperlipidemia induced by HFD (Supplementary Fig. S16A–H). Compared to HFD mice, leptin-deficient *ob/ob* mice are models of massive metabolic dysfunction, developing severe obesity, dyslipidemia, and hepatic steatosis early in life [88]. We similarly administered MgUGN to *ob/ob* mice over the course of 14 days. The treatment markedly reduced hepatic lipid droplets (Fig. 7A and Supplementary Fig. S16I–J), both in size and abundance, without altering body weight or food intake (Fig. 7B–C). Liver weight and hepatic as well as plasma triglyceride (TG) levels were also lower in MgUGN-treated *ob/ob* mice than in controls (Fig. 7D–F), with similar reductions observed for plasma total cholesterol (TC) and LDL-C levels (Fig. 7G and Supplementary Fig. S16K). These results together indicate that MgUGN treatment can alleviate both diet and genetic obesity-associated hepatic steatosis and dyslipidemia, underscoring its therapeutic potential for chronic metabolic diseases.

We next asked whether the metabolic benefits of MgUGN could translate into better resilience during acute hepatotoxic stress. To test this, we used an acetaminophen (APAP) overdose model and evaluated acute liver damage (ALI) 24 h after challenge. Given that MgUGN reached peak liver distribution within 2 h (Supplementary Fig. S15A–B), we administered MgUGN intravenously 2 h prior to intraperitoneal injection of APAP (Fig. 7H). Serum aminotransferase levels (ALT and AST) rose sharply after APAP administration (Fig. 7I–J), reflecting hepatocellular injury. Pretreatment with MgUGN significantly blunted this increase, indicating reduced liver damage. These biochemical results were supported by histological analysis (Fig. 7K–L). The APAP-only group showed widespread necrosis and disorganized hepatic cords, while MgUGN-treated mice retained clear, orderly hepatic architecture with no obvious sinusoidal collapse, hemorrhage, or marked degenerative changes. TUNEL staining revealed the expected rise in apoptotic cells in the APAP group, and this signal was substantially reduced by MgUGN pretreatment (Fig. 7M–N). Furthermore, hepatic levels of TNF-α and IL-1β, which rise sharply during APAP-triggered inflammatory responses, returned to near-baseline in MgUGN-treated mice (Fig. 7N–P). This anti-inflammatory effect further demonstrates that MgUGN prevents the progression of MASLD into liver fibrosis, cirrhosis, and liver failure under inflammatory conditions. Taken together, our *in vitro* and *in vivo* data suggest a broad ability of MgUGN to restore liver homeostasis and identify ROAM1 as a potential therapeutic target for metabolic liver disease and for preventing toxin-induced ALI.

### 2.9 Discussion

In this study, we identified UDP-GlcNAc as a specific lysosomal AMPK activator that primarily controls intracellular lipid metabolism. When UDP-GlcNAc assembled into nanosheets (MgUGN), it showed good bioavailability and prolonged AMPK activation, efficiently treating NAFLD in HFD-fed and *ob/ob* mice. Mechanistically, UDP-GlcNAc acts through the lysosomal protein ROAM1 to activate lysosomal AMPK, which then selectively phosphorylates the lipogenic substrate ACC1 and represses Srebp1c and Fasn expression, thus suppressing lipid synthesis and reducing fat content *in vivo* (Graphical Abstract).

UDP-GlcNAc is the end product of the HBP, a nutrient-sensing hub, and connects nutrient availability to protein glycosylation, especially O-GlcNAcylation, and downstream signaling [52,89]. Higher UDP-GlcNAc and HBP flux have also been linked to insulin resistance and leptin expression, where elevated UDP-GlcNAc in muscle markedly impairs insulin responsiveness [90,91] and increased tissue UDP-GlcNAc rapidly upregulates leptin mRNA and protein expression [91], highlighting its role as a metabolic regulator. While emerging evidence suggests that UDP-GlcNAc, largely through O-GlcNAcylation, intersects with AMPK signaling, whether UDP-GlcNAc is a physiologically relevant regulator of AMPK and how tissue- or compartment-specific effects influence AMPK signaling have not been established. In this study, we show that UDP-GlcNAc-induced AMPK activation occurred without detectable changes in bulk O-GlcNAcylation, and its hepatoprotective effects were preserved in leptin-deficient mice, arguing against classical HBP-to-O-GlcNAc or leptin-dependent mechanisms. Instead, we observed activation of lysosomal AMPK mediated through ROAM1 scaffolding, consistent with a model in which UDP-GlcNAc promotes lysosome-centered AMPK signaling and liver resilience. With respect to lipid metabolism, UDP-GlcNAc effectively reduces hepatic fat by activating AMPK and upregulating leptin levels, highlighting its strong potential as a therapeutic agent for MASLD. While in-depth analysis of this model is beyond the scope of the paper, further studies of liver or lysosome-specific disruption of AMPK signaling should greatly facilitate our understanding of how AMPK orchestrates responses to organelle and tissue-specific cues.

It is becoming evident that spatially distinct pools of AMPK are differentially activated depending on local cues [92]. In the model of hierarchical activation, lysosomal AMPK is often activated earliest during nutrient shortage [25,27,93]. For instance, lysosomal AMPK activity can be modulated by fructose-1,6-bisphosphate via the LKB1-Axin complex and activated AMPK selectively phosphorylate lysosome localized and metabolic substrate [27]. Here, we demonstrate that UDP-GlcNAc activates AMPK on lysosomes, initiating a signaling cascade that spreads to other compartments. This spatiotemporal pattern is attributable to ROAM1. Several SLC35 proteins (e.g., SLC35A3/A4/D2) localize to and function in the Golgi apparatus [[51], [52], [53]]. In contrast, while ROAM1 can be found on the Golgi apparatus and plasma membrane, it primarily localizes to lysosomes, which enables ROAM1 to interact with AMPKβ subunits and repress lysosomal AMPK activity. Our data indicate that the ROAM1 TM domain, conserved among SLC35 family members for UDP-sugar binding [50], is required for ROAM1 function. The UDP-GlcNAc–ROAM1–lysosomal AMPK axis preferentially phosphorylates metabolic substrates, particularly the lipogenic protein ACC1 rather than the lipolytic protein ACC2, thereby selectively suppressing lipid synthesis and reducing intracellular fat content. Unlike A769662, UDP-GlcNAc specifically and efficiently inhibits ACC1 to regulate lipid metabolism without affecting other AMPK-mediated processes such as mitochondrial function. These observations further support the notion that lysosomal AMPK activation is more favorable for the regulation of lipid metabolism.

As a central hub for coordinated anabolic and catabolic responses to energy and nutrient cues, the lysosome is also where AMPK and mTORC1 signaling converges [94,95]. Under low-energy conditions, lysosome-localized AMPK is activated through the AXIN–LKB1 complex and suppresses mTORC1 by phosphorylating key components of the Rag and Raptor pathways [96]. Under nutrient-rich conditions, the Rag GTPases and Ragulator recruit mTORC1 to the lysosome, favoring growth and biosynthesis [97]. Several solute carrier proteins, including SLC38A9, SLC7A5, SLC3A2, and PAT1, have been implicated in mTOR regulation during amino acid starvation [[98], [99], [100]]. It is conceivable that elevated AMPK activity at the lysosome, as a result of UDP-GlcNAc stimulation or ROAM1 inhibition, could influence mTORC1's access to nutrients, its residency time, or its phosphorylation state, which in turn coordinates nutrient sensing and the switch to conservation mode when energy drops. Further research into whether and how UDP-GlcNAc-ROAM1 signaling intersects with mTORC1 should shed more light on the dynamic and compartmentalized control of metabolic responses.

In this study, MgUGN was designed not as a complex multifunctional nanocarrier, but as a simple, biocompatible, and efficient delivery platform to overcome the pharmaceutical limitations of UDP-GlcNAc, including poor membrane permeability and rapid clearance. Mg^2+^ was selected as the coordinating ion due to its physiological abundance, excellent biocompatibility, and minimal toxicity. Through coordination interactions between Mg^2+^ and the phosphate groups of UDP-GlcNAc, the metabolite self-assembles into stable nanosheets. Control experiments confirmed that neither Mg^2+^ alone nor free UDP-GlcNAc produced significant effects, whereas MgUGN elicited robust AMPK activation and superior therapeutic efficacy, demonstrating that the observed bioactivity arises from the magnesium-coordinated nanosheet architecture that enables efficient delivery and sustained release.

Our results from MgUGN-treated HFD-fed mice, leptin-deficient *ob/ob* mice, and APAP-induced ALI mice underscore the versatility of the UDP-GlcNAc-ROAM1-AMPK axis and position UDP-GlcNAc/ROAM1 as potential therapeutic targets for MASLD. UDP-GlcNAc provides a potent, ROAM1-dependent route to activate AMPK *in vivo*. In metabolic disease models, UDP-GlcNAc-driven AMPK activation improves lipid handling and reduces hepatic steatosis, even in the absence of leptin signaling, which points to its broader application potential, particularly for metabolic liver diseases with impaired adipokine signaling. In the APAP ALI model, prophylactic MgUGN treatment preserves hepatic architecture and reduces cell death, consistent with rapid AMPK-dependent stress adaptation. These outcomes support a shared mechanism in which MgUGN activates lysosomal AMPK to stabilize metabolic and cellular homeostasis. Given that UDP-GlcNAc/ROAM1 appear to regulate AMPK in a localized manner, MgUGN may also allow for selective enhancement of protective pathways with lower risks of unwanted global effects. Further experiments on upstream cues that control UDP-GlcNAc/ROAM1 interaction and pharmacokinetic parameters should help advance its translational applications.

## 3 Materials and methods

### 3.1 Plasmids

cDNAs encoding AMPKα1, AMPKα2, AMPKβ1, AMPKβ2, AMPKγ1, OMP25, TMEM192, SLC35F2, SLC35F3, SLC35F5 and SLC35F6 (ROAM1) were cloned into pDONR or pEntry vectors and then Gateway cloned into destination vectors including pDEST27 (Invitrogen), plenti-SFB (EF1α promoter and c-terminal Flag tag) [101], plenti-HA (EF1α promoter and N-terminal HA tag)and plenti-Myc vector (EF1α promoter and N-terminal Myc tag).

### 3.2 siRNAs

Nucleotide sequences of siRNAs used in the study are as follows:

Human *ROAM1*:

5′-GCATGGTGTTGGACAGCTT-3′(#1),

5′-GCATTGCCTTCTTCAACTT-3′ (#2),

5′-GAGGAGAAGTTCGTCTACA-3′(#3),

Human *SLC35D1*:

5′-CGAAACGCTGACCGTGTTT-3′(#1),

5′-GCACGCAGTATAATTCTGC-3′(#2),

5′-GACAGAAATGTACCTCGAA-3′(#3),

Human *SLC35A3*:

5′-GAAGGACCTCGTTATCTAT-3′(#1),

5′-CAGGTCACGTATCAGTTAA-3′(#2),

5′-GCGTTATTCCAGAACTTTA-3′(#3).

Human *UAP1*:

5′-AGACGTCTTGGACAACTGATT-3′(#1),

5′-AGTAGCAGTTCTTCTTCTATT-3′(#2),

Mouse ROAM1:

5′-GAGGAGAAGTTTGTCTACA-3′.

### 3.3 Reagents

Antibodies against pAMPKα-T172 (#2535), AMPKα (#2532), pACC-S79 (#11818), ACC (#3676), ULK1(#8054), pULK1-S555(#5869), Raptor(2280), pRaptor- S792(2083), LKB1 (#3047), AMPKβ1 (#4178), AMPKβ2 (#4148), LAMP1(#15665), GM130 (#12480), Flag (14793S), HA (#3724), pTSC2 (#2280), TSC (#4308), O-GlcNAc MultiMab® (#82332), pMFF-S146 (#49281), MFF (#86668), VDAC (#4661), ACC1 (#4190), ACC2 (#8578), GST (#2622), pYAP-S127 (#13008S) and BAY-3827 (#60887) were purchased from Cell Signaling Technology. Anti-UAP1 antibody (# 67545-1-Ig) was purchased from Proteintech. Antibodies against the Flag tag (F7425), SLC35F6/ROAM1 (HPA034655) (for IF), the Myc epitope (M4439), OSMI-1 (SML1621), and α-Tubulin (T5168) as well as anti-Flag M2 Affinity Gel (A2220) and D-Glucosamine (G4875) were purchased from Sigma. Antibodies against SLC35F6/ROAM1 (for WB) (GTX46320) were purchased from GeneTex. Goat anti-rabbit Alexa-488 (A-21206) and goat anti-mouse Alexa-555 (A31570) were purchased from Invitrogen. Goat anti-rabbit IRDYE-800 (926-32211) and goat anti-mouse IRDYE-680 (926-68072) were purchased from LI-COR. Glutathione Sepharose™ 4B resin (17075605) was from Cytiva. Additional reagents include the CAMKK2 inhibitor STO-609 (S275918, Aladdin), AICAR (2840 Tocris), and Pierce™ Protein A/G Agarose (20422). L-Glutathione reduced (1294820), MitoTracker™ Red FM (M22425), and BODIPY™ 493/503 (D3922) were purchased from Thermo Fisher Scientific. The 3xFlag Peptides (A6001) were purchased from APExBIO. Uridine 5′-Diphospho-N-acetylglucosamine Disodium Salt (UDP-GlcNAc) was obtained from Shanghai Macklin Biochemical Co., Ltd.

### 3.4 Cell culture and treatment

HEK293T, HTC75, HepG2, and MEF cells were cultured in DMEM (Gibco, 11,875-093) supplemented with 10% fetal bovine serum (FBS) (Excell biotech, FSP500). Purchased AML12 cells (iCell, iCell-m003) were grown at 37°C in designated media (iCell, iCell-m003-001b). All cell lines were maintained at 37°C in a humidified incubator with 5% CO_2,_ and confirmed to be mycoplasma free. Cell lines stably expressing various proteins were established through lentivirus transduction as previously described [102].

For glucose starvation, cells were washed twice with 1xPBS and then incubated in glucose and pyruvate-free DMEM (Invitrogen) supplemented with 10% FBS. Plasmids and siRNAs were transfected using Polyethylenimine (Polyscience, 23,966) and Lipofectamine RNAiMAX (Thermo, 13778150) respectively.

For UDP-GlcNAc electroporation, ∼1x10 [6] cells were collected by centrifugation at 90 x *g* for 8min and resuspended in 20 μL working solution containing UDP-sugars at the desired concentration. The working solution was a 1:50 mixture of Solutions Ⅰ (0.36 M ATP·disodium salt, 0.59 M MgCl_2_·6H_2_O) and Ⅱ (88 mM KH_2_PO_4_, 14 mM NaHCO_3_, 2 mM Glucose, pH7.4). The cell suspension was then transferred to a sterile 0.2-cm cuvette and electroporated using a Lonza 4D-Nucleofector System (DS150 program). Immediately after electroporation, 80 μl complete media was added for a 40-min recovery, after which cells were cultured in 2 ml of complete medium for the required duration before further analysis. For glucosamine (GlcN) treatment, cells were serum starved for 4 h before GlcN was added [103].

### 3.5 Genome-wide bi-molecular fluorescence complementation (BiFC) screens

HTC75 cells stably expressing the hORFeome prey library with ∼18,000 full-length YFPc-tagged human ORFs were described previously [31]. For the bait, the cDNAs encoding AMPKβ1 and AMPKβ2 were tagged with YFPn on either terminus and used to generate retroviruses for the HTC75 prey library cells library, followed by G418 (250 μg/ml) selection for 10 days. HTC75 cells expressing YFPn-AMPKβ1 and β2 were used as negative controls. YFP-positive cells were then FACS sorted, expanded in culture, and enriched by four rounds of FACS sorting, until >90% of the cells were YFP positive. Total RNA was then extracted for RT-PCR and next-gen sequencing as described before [32].

### 3.6 Inducible ROAM1 knockout (KO) cells

HEK293T cells stably expressing tetracycline-inducible Cas9 (iCas9 cells) were generated as previously described [104,105], and individual cell clones with robust doxylcycline-inducible Cas9 expression were selected for further experiments. Three gRNAs targeting different regions of SLC35F6/ROAM1 exon 1 were cloned into the LentiGuide vector [104] for subsequent lentiviral packaging and transduction of iCas9 cells. Pooled inducible ROAM1 KO cell lines were generated by culturing iCas9 cells stably expressing the individual gRNAs in doxylcycline (1 μg/mL) for 3 days. Cells expressing gRNA #2 were also used to stably express full-length or deletion mutations of ROAM1. These rescue expression cells were maintained in doxycycline to ensure ROAM1 KO.

gRNA1 of *ROAM1*: 5′-CCCATGTACTACATCCCCGC-3′,

gRNA2 of *ROAM1*: 5′-AGGCCATGTCGGCGGACGCT -3′,

gRNA3 of *ROAM1*: 5′-CGGCTCCATCAACACGCTCT -3′

### 3.7 Mouse studies

All animal procedures were reviewed and approved by the Institutional Animal Use and Care Committee of Sun Yat-sen University and conducted in accordance with institutional guidelines. All efforts were made to minimize suffering.

• Mouse strains and husbandry

Male C57BL/6 J mice aged 8-10 weeks were purchased from Guangzhou Gem Pharmatech Animal Company. Eight-week-old male *ob/ob* and C57BL/6 J mice were purchased from Cyagen Biosciences. Mice were housed in a specific pathogen–free facility at 21 ± 1°C with humidity of 55% ± 10% and a 12-h light/dark cycle. Animals were kept in ventilated cages with standard bedding, provided chow and water ad libitum, and given basic enrichment.

• ROAM1 knockdown using siRNAs

siRNAs were injected into the muscle and liver (2 nmol/100 g) of male C57BL/6 J mice aged 8-10 weeks using the Entranster-in vivo for RNA kit (18668-11-1, Engreen Biosystem). Injections were administered into liver on day1 or the same muscle region on days 1 and 3. Three days after liver injection or two days after the muscle second injection, tissues were collected and stored at −80°C. The following siRNAs (Igebio) were used: siCtrl: 5′-UUC UCC GAA CGU GUC ACG UTT-3′. siROAM1: 5′-GAG GAG AAG TTT GTC TAC A-3′.

• *MgUGN biodistribution analysis by fluorescence imaging*

Fifteen 8-week-old male C57BL/6 mice received equivalent doses of Cy5.5-labeled MgUGN nanosheets dispersed in 1x PBS via intravenous tail injection. Major organs (heart, liver, spleen, lung, and kidney) were collected at 1, 3, 6, 12, and 24 h post injection (n = 3 per time point), imaged (ex:660 nm and em:710 nm) using a small-animal imaging system (IVIS Spectrum, PerkinElmer), and quantified for fluorescence intensity.

• MgUGN treatment in different mouse models

For *in vivo* toxicity assessment, eight-week-old male C57BL/6 mice were randomly assigned to three groups (n = 6 per group). For intravenous injections, Group 1 (control) received 1× PBS (200 μL), and Groups 2 and 3 received MgUGN (10 mg/kg, 200 μL). Blood and major organs (heart, liver, spleen, lungs, and kidneys) were collected from Group 2 after seven days and Groups 1 & 3 after 14 days. For the high-fat diet (HFD) model, male C57BL/6 J mice aged 8-weeks were fed a HFD (60 kcal% fat, D12492, Research Diets) for 12 weeks. To evaluate MgUGN, *ob/ob* and HFD-fed mice were randomized into control and treatment groups (n = 6 per group), and administered MgUGN (10 mg/kg) via tail vein injection every 72 h for two weeks. For the acute acetaminophen (APAP) overdose model, eight-week-old male C57BL/6 mice were randomly assigned (n = 6 per group) to groups #1 (healthy), #2 (APAP model), and #3 (APAP + treatment) and placed on a 16-h fast. Group #1 received an intraperitoneal injection of 1xPBS. Groups 2 & 3 were intravenously administered respectively 1xPBS and MgUGN (10 mg/kg, 200 μL) via the tail vein 2 h before APAP administration (250 mg/kg in saline). After 24 h, blood and liver samples were collected for further analysis. To assess AMPK activation, C57BL/6 J mice received a single injection of MgUGN (10 mg/kg) and liver samples were collected 36 h later for Western blot analysis.

• Analysis of mouse blood and tissue samples

Hematological parameters were analyzed using an automated hematology analyzer (BC-5000 Vet), including white blood cells (WBC), lymphocytes (LYM), monocytes (MON), neutrophils (NEU), eosinophils (EOS), basophils (BAS), red blood cells (RBC), hemoglobin (HGB), hematocrit (HCT), mean corpuscular hemoglobin (MCH), platelets (PLT), and plateletcrit (PCT). Serum was obtained by centrifugation of clotted blood at 2000 × g for 15 min at 4°C. Commercial kits from Beyotime were used to assay hepatic and renal function biomarkers alanine aminotransferase (ALT)(P2711S), aspartate aminotransferase (AST)(P2715S), blood urea nitrogen (BUN)(S0574S), and creatinine (CREA)(S0291), as well as plasma levels of triglycerides (TG) (S0219S), total cholesterol (TC) (S0211S), and LDL-cholesterol (S0213S).

Tissue samples were resuspended in appropriate buffers and homogenized using a Bullet Blender Tissue Homogenizer (Next advance) on ice with 8 cycles of 1min on and 5min off. For glucose, Tissue Lysis Buffer (S3062, Beyotime) (30 mg/300 μL) and the Glucose Assay Kit with O-toluidine (S0201S, Beyotime) were used. For hepatic and cellular TG, liver tissues (approximately 100 mg) and cell lysates were homogenized in 1 mL of 5% NP-40, followed by heating at 95°C for 2-5 min and centrifugation at 16,000 × g for 15 min. For malonyl-CoA and proteins, tissues were lysed in buffer containing 140 mM NaCl, 50 mM Tris-HCl, pH 8.0, and 1% Triton-X100. The Malonyl-CoA ELISA kit (S203685B, Shanghai Dibai Biotechnology Co., Ltd) was used to quantify malonyl-CoA. For triglycerides, extraction buffer (5% NP-40 in water) and the High Sensitivity Triglyceride Fluorometric Assay Kit (MAK264, Sigma) were used. RNA was extracted using Trizol (Invitrogen). Proteins and RNA were analyzed by western blotting and qPCR respectively.

Histopathological assessment of organ tissues was performed using hematoxylin and eosin (H&E) staining. Liver tissues were fixed in 4% formalin and embedded in paraffin. Consecutive sections were stained with H&E to assess steatosis. TUNEL staining (C1086, Beyotime) and immunofluorescence staining (TNF-α and IL-1β) (AB2026 and AB2038, Beyotime) were also performed. For neutral lipid visualization, frozen sections were stained with Oil Red O (Sigma). All images were analyzed and quantified using ImageJ (v.1.53c), with regions of interest selected to exclude major vessels and liver capsule. Oil Red O staining was quantified as the ratio of Oil Red O-positive area to total tissue area. Histologic scoring included assessment of inflammation and necrosis and performed on randomly assigned samples by investigators blinded to group allocation. For lobular inflammation, scores were assigned as follows: 0 for none, 1 for <10% of tissue, 2 for 10–30%, 3 for 30–50%, and 4 for >50%. Necrosis was scored as 0 for none, 1 for <10% of tissue, 2 for 10–20%, 3 for 20–30%, and 4 for >30%. Inflammation and necrosis scores were then summed to generate the total histologic score. For H&E staining related to ALI, The area of the lesion region was quantified as a percentage of the total tissue area. For TUNEL, TNF-α and IL-1β staining, the proportion of cells with positive signals relative to the total cell count was calculated.

### 3.8 Immunoprecipitation (IP), GST pulldown, and western blotting

Cell lysis, IP, GST pulldown, and western blotting were carried out essentially as previously described [24]. Briefly, cells were lysed in lysis buffer (50 mM HEPES at pH 7.5, 150 mM NaCl, 1 mM EDTA, 1% NP-40, 10 mM pyrophosphate, 10 mM beta-glycerophosphate, 50 mM NaF, 1.5 mM Na_3_VO_4_, 1 mM dithiothreitol (DTT), and the protease inhibitor cocktail (Roche)) for 30 min and centrifuged at 12, 000 g for 15 min. To better solubilize membrane proteins such as ROAM1, the nonionic detergent n-Octyl beta-D-Glucopyranoside (ODG) was also added (2%) whenever necessary. Whole cell extracts were incubated with anti-Myc antibodies (1:100) overnight at 4°C followed by protein A/G agarose beads for 2 h at 4°C for anti-Myc IP, or with anti-Flag M2 beads (1:20) for 2 h at 4°C for anti-Flag IP, or with Glutathione Sepharose 4B beads (Cytiva, 17075605) for 2 h at 4°C for GST pulldown. For endogenous co-IP assays, mouse liver tissue lysates were incubated with the indicated antibodies overnight at 4°C, followed by capture with protein A/G agarose beads. Immunoblotting was analyzed using an Odyssey infrared imaging system (LI-COR Biosciences).

### 3.9 Immunofluorescence, lipid droplet, and Oil Red O staining

For immunofluorescence staining, cells were seeded on 15 mm glass coverslips at 30% confluence. After 24hrs, cells were washed with 1×PBS and fixed in 4% fresh paraformaldehyde at 4°C for 10min. Then, cells were permeabilized (5% Triton-X, 20 mM HEPES, 3 mM MgCl_2_, 300 mM Sucrose) 15min and blocked (5% goat serum in PBS) 1 h at room temperature. After incubation with primary antibodies overnight, the coverslips were washed and incubated with appropriate secondary antibodies for 1 h. After 3 × 10 min washes, cells were stained with DAPI and mounted for visualization under a confocal microscope (Leica). Fluorescence images were quantified with the LASX software. For lipid droplet staining, cells at ∼70% confluence gon coverslips were incubated with BODIPY 493/503 (20 μg/ml) for 10 min at 37°C, fixed in 4% paraformaldehyde at 4°C for 10 min, and counterstained with DAPI. Images were acquired on a Leica confocal microscope and processed using LASX and ImageJ. For Oil Red O staining, cells at 60% confluence on coverslips were fixed with 4% paraformaldehyde solution for 10 min at 4°C, washed three times with distilled water, and stained with Oil Red O (MA0120; meilunbio) for 1 h before being washed five times The dye was then eluted with 60% isopropanol for 2min, and images were captured with a Leica microscope and processed in ImageJ.

### 3.10 Reverse transcription and qPCR

Total RNA was extracted using Trizol (Invitrogen) and phenol-chloroform for cDNA synthesis using the PrimeScript™ RT Reagent Kit (TaKaRa). qPCR was performed with HiScript® II Q RT SuperMix for qPCR (Vazyme) on a qTOWER3 system (Analytikjena). Gene expression was quantified using the ΔΔCt method with GAPDH as the control. The following primers were used in this study:

Human:

*GAPDH* forward primer (FP): 5′-GGAGCGAGATCCCTCCAAAAT-3′

*GAPDH* reverse primer (RP): 5′-GGCTGTTGTCATACTTCTCATGG-3′

*SLC35D1* FP: 5′-ATCGTGGTGGTGAATAAGAGCG-3′

*SLC35D1* RP: 5′-GGAAACGTCTTTCGAGGTACATT-3′

*SLC35A3* FP: 5′-CAGTGGCTGTCCCTAGTAATTTT-3′

*SLC35A3* RP: 5′-AGAACTGCCATGAGTCCTACA-3′

Mouse:

*Srebp1c* FP: 5′-GCAGCCACCATCTAGCCTG-3′

*Srebp1c* RP: 5′-CAGCAGTGAGTCTGCCTTGAT-3′

*Fasn* FP: 5′-GCTGCGGAAACTTCAGGAAAT-3′

*Fasn* RP: 5′-AGAGACGTGTCACTCCTGGACTT-3′

*Atgl* FP: 5′-ATGTTCCCGAGGGAGACCAA-3′

*Atgl* RP: 5′-GAGGCTCCGTAGATGTGAGTG-3′

*Hsl* FP: 5′-GATTTACGCACGATGACACAGT-3′

*Hsl* RP: 5′-ACCTGCAAAGACATTAGACAGC-3′

*Cd206* FP: 5′-CTCTGTTCAGCTATTGGACGC-3′

*Cd206* RP: 5′-TGGCACTCCCAAACATAATTTGA-3′

*Cd301* FP: 5′-CAATGTGGTTAGTTGGATCGGC-3′

*Cd301* RP: 5′-CCCAGTTCTTAAAGCCTTTCTCA-3′

PGC1α FP: 5′-TATGGAGTGACATAGAGTGTGCT-3′

PGC1α RP: 5′-GTCGCTACACCACTTCAATCC-3′

ERRs FP: 5′-GGGGAGCATCGAGTACAGC-3′

ERRs RP: 5′-AGACGCACACCCTCCTTGA-3′

### 3.11 Cellular ATP and AMP content analysis by LC-MS

For LC–MS, ∼1x10 [6] cells were sonicated at 30% Ampl for 30min (Sonics Vibra-Cell; VCX130PB) in 100 μL methyl alcohol, followed by an addition of 100 μL water and vortexing for 10 min. After centrifugation at 12,500 g for 10 min at 4°C, the supernatant was mixed with water in equal volume. Measurement of ATP, ADP and AMP was performed using a XEVO TQ-S Micro mass spectrometer (Waters Corporation) interfaced with an ACQUITY UPLC I-Class PLUS System (Waters Corporation), equipped with the Waters UPLC BEH C8 column (1.7 μm, 2.1 mm × 100 mm). Mobile phase A consisted of 0.05% (v/v) ammonia-water and 0.5 mg/mL ammonium acetate in water. Mobile phase B was methanol. The column was maintained at 45°C and equilibrated with 0.5% buffer B for 1 min at a flow rate of 0.3 ml/min. Aliquots of 1 μL of each sample were injected and compounds were eluted with a linear gradient of 0.5-40% buffer B over 3 min. Buffer B was then increased to 90% in 0.5 min, followed by a 5min wash at 90%. Eluents were directed into the mass spectrometer with the ion source set to 150°C and the vaporizer at 500°C. The instrument was operated in positive mode with source voltage at 3000 and sheath gas at 20. Levels of ATP and AMP were quantified using the TargetLynx quantification software.

### 3.12 In vitro incubation of cell lysates with UDP-sugars

Cells (1 × 10^6^) were washed once with ice-cold low-salt ODG buffer (50 mM Tris-HCl at pH7.5, 10 mM NaCl, 1 mM EDTA, 1 mM EGTA, 5 mM β-Me, 2%ODG) and once with ice-cold high-salt ODG buffer (50 mM Tris-HCl at pH7.5, 50 mM NaCl, 1 mM EDTA, 1 mM EGTA, 5 mM β-Me, 2%ODG) plus protease inhibitor cocktail (Roche). Cells were then lysed with a 7-mL Dounce homogenizer (120 strokes) at 4°C. Following centrifugation at 1000 x *g* for 10 min at 4°C, the supernatant was collected for incubation with various UDP-sugars for 1hr at 4°C. The samples were then boiled in 5×SDS loading buffer and analyzed by western blotting.

### 3.13 In vitro phosphatase assays

For *in vitro* phosphatase assays, bound proteins were first eluted from Flag beads, protein A/G beads, or HA beads with the 3xFlag peptide (250μg/ml) (APExBIO, A6001) or HA peptide (250μg/ml) respectively. The eluted proteins were then incubated in the phosphatase assay buffer (100 mM Tris-HCl, 100 mM NaCl, 2 mM MgCl_2_, 1.5 mM Zn_2_SO_4_ and protease inhibitor cocktail (Roche)) at 37°C for 1 h before western blotting.

### 3.14 Detection of UDP-GlcNAc by ELISA

For the ELISA assay, ∼1x10 [7] cells were resuspended in 300ul ice-cold 1xPBS and sonicated at 30% Ampl for 5 cycles of 3s on and 7s off (Sonics Vibra-Cell; VCX130PB) before centrifugation at 350 x g for 20 min at 4°C. Supernatant was collected for use with the UDP-GlcNAc ELISA Kit (MM-2354H2; MEIMIAN). Absorbance at 450 nm was detected on a Synergy HTX plate reader (BioTek).

### 3.15 Assembly of MgUGN nanosheets and Cy5.5 labeling

A 5 mL solution of 20 mM magnesium chloride hexahydrate (MgCl_2_·6H_2_O) (Shanghai Aladdin Biochemical Technology Co., Ltd) was added to 5 mL of 10 mM UDP-GlcNAc under constant stirring at room temperature. After 30 min, 40 μL of 5 M sodium hydroxide (NaOH)(Shanghai Aladdin Biochemical Technology Co., Ltd) was added to induce formation of a faint white precipitate, and the mixture was stirred for another 2 h. The resulting nanosheets were collected by centrifugation at 12,500 x g for 5 min, washed three times with deionized water, freeze-dried, and stored at 4°C until use.

For Cy5.5 labeling, 10 mg of MgUGN nanosheets was added to 2 mL of an aqueous solution containing 1 mg Sulfo-Cyanine5.5 (Cy5.5)(MedChemExpress). The mixture was sonicated for 5 min, then magnetically stirred at 4°C for 12 h. Subsequently, the Cy5.5-MgUGN nanosheets were collected by centrifugation at 12,500 x g for 5 min, washed three times with deionized water, and finally re-dispersed in deionized water.

### 3.16 Characterization of nanosheets

The morphology and elemental distribution of nanosheets were characterized using transmission electron microscopy (TEM, FEI Talos F200S, 200 kV). The hydrodynamic diameter and surface zeta potential of nanosheets dispersed in deionized water were measured using a NanoBrook 90Plus PALS analyzer (Brookhaven Instruments, USA). Fourier transform infrared (FTIR) spectra were obtained using a Bruker VERTEX 70 spectrometer. An X-ray photoelectron spectrometer (XPS, Thermo Fisher Scientific Nexsa) was employed to analyze the full survey spectrum and high-resolution spectra of Mg 1s, O 1s, C 1s, N 1s, and P 2p. X-ray diffraction (XRD) patterns were collected using a Bruker D8 Advance diffractometer equipped with a Cu Kα radiation source.

### 3.17 Loading capacity, content release, and cellular uptake properties of MgUGN

To measure loading capacity, freeze-dried nanosheets were completely dissolved in 1 M HCl before analysis using UV-Vis spectroscopy. UDP-GlcNAc content was determined based on comparing absorbance at 262 nm to a standard curve. The monitor UDP-GlcNAc release, 3 mg of freeze-dried MgUGN nanosheets were dispersed in 5 mL of 1xPBS at different pH (7.4, 6.5, and 5.5)(n = 3 per group) at 37°C under gentle shaking (100 rpm). At predetermined time points (1, 3, 6, 12, 24, 48, and 72 h), 1 mL supernatant was collected after centrifugation at 500 x g for 5 min, and an equal volume of fresh 1xPBS was added back. The UV-Vis absorption spectra of the collected samples were measured on a Lambda 365+ UV-Vis spectrophotometer (PerkinElmer). The cumulative release of UDP-GlcNAc was calculated from absorbance at 262 nm. For cellular uptake, 1 × 10^5^ cells were seeded in 24-well plates and cultured overnight in 500 μL of DMEM with 10% FBS. The medium was then replaced with 500 μL of fresh media containing Cy5.5-MgUGN (100 μg/mL). Live-cell imaging was performed to capture bright-field images and fluorescent signals using a Leica confocal microscope and images were processed using LASX and ImageJ.

### 3.18 Molecular Docking and Molecular Dynamics Simulation

The AlphaFold-predicted structure of SLC35F6 (UniProt: Q8N357) was downloaded from the UniProt database. The three-dimensional structure of UDP-GlcNAc (CID: 445675) was obtained from the PubChem database in SDF format. Molecular docking between SLC35F6 and UDP-GlcNAc was performed using AutoDock Vina (version 1.5.7). The receptor and ligand were prepared by adding polar hydrogens and assigning Gasteiger charges. A global docking search was conducted within a grid box encompassing the entire protein surface. The binding mode with the lowest binding free energy was selected for subsequent molecular dynamics (MD) simulations. Visualization and structural analysis of the docking poses were carried out using PyMOL (Schrödinger, Inc.).

Molecular Dynamics Simulation: MD simulations were performed using GROMACS 2022.2 to evaluate the dynamic stability of the SLC35F6-UDP-GlcNAc complex. The receptor topology was generated using the pdb2gmx module with the Amber99SB-ILDN force field. Ligand topology and parameters were built using the GAFF2 force field via the sobtop program, and atomic partial charges for the ligand were assigned using the RESP method. The complex was solvated in a cubic box with TIP3P water molecules, maintaining a minimum distance of 1.0 nm between the protein and the box edges. Na^+^ and Cl^−^ ions were added to achieve a physiological ionic strength of 0.15 M. Energy minimization was performed using the steepest descent and conjugate gradient methods, followed by 10 ps NVT and 10 ps NPT equilibration at 300 K and 1 bar with position restraints on the protein and ligand. The production run was carried out without restraints for 100 ns. Long-range electrostatic interactions were treated using the Particle Mesh Ewald (PME) method, with a cutoff of 1.2 nm for van der Waals and short-range electrostatic interactions. All bonds were constrained using the LINCS algorithm, and the integration time step was 2 fs. The resulting trajectory was analyzed using GROMACS utilities: root-mean-square deviation (RMSD) to assess overall structural stability, root-mean-square fluctuation (RMSF) to evaluate local residue flexibility, radius of gyration (Rg) to examine protein compactness, solvent-accessible surface area (SASA) to monitor protein-solvent interactions, and hydrogen bond occupancy to characterize intermolecular interactions. Principal component analysis (PCA) coupled with the gmx sham tool was used to construct the free energy landscape (FEL) and identify the most stable conformational states of the complex.

### 3.19 Statistical analysis

Statistical analyses were carried out with Excel or GraphPad Prism version 8.0. The number of independent biological repeats (n) as well as error bars and mean (S.E.M) are indicated in the figure legends. A two-tailed *t*-test and a one-way ANOVA test were used to assess the *P* value.

## Ethics approval and consent to participate

All animal experiments were ethically approved by the Institutional Animal Care and Use Committee (IACUC) of Shenzhen Lingfu Top Biotech Co., Ltd. (Approval ID: TOPGM-IACUC-2024-0377, approved on 27 June 2025). All animal operations followed institutional SOPs and the 3R principles of laboratory animal welfare. Ninety male SPF C57BL/6 mice (8 weeks old) sourced from Cyagen Biosciences were used in barrier facilities. Mice were anesthetized before injection and modeling procedures, and sacrificed by carbon dioxide asphyxiation at experimental endpoints. All animal carcasses were treated via standardized harmless disposal. All experimenters held valid animal operation training certificates, and veterinary supervision was provided throughout the study. No human participants or human biological materials were involved in this work.

## Funding

This work was funded by grants from the 10.13039/501100001809National Natural Science Foundation of China (32330023 to Z.S., 32170757, 82371563, 92249304 to W.M., 82302281 to T.S., 32470794 and 32071433 to F.L.), Basic and Applied Basic Research of Guangdong Province (2021B1515140056 to Songbo Li), Shenzhen Science and Technology Program (Grant No. JCYJ20240813150311015 and JCYJ20230808110006013 to T.S.), a Novel Radiation-Free Navigation System (Grant No. XMHT20240115001 to F.W.).

The authors thank Dr. Jun Cui for supplying antibody and siRNA of UAP1, Dr. Dan Liu for her excellent work on manuscript revision.

## CRediT authorship contribution statement

**Jianxi Zhu:** Data curation, Investigation, Writing – original draft, Writing – review & editing. **Tuanwei Sun:** Investigation. **Fuxin Wei:** Investigation. **Dong Wang:** Investigation. **Feng Liu:** Funding acquisition, Supervision. **Dongming Chen:** Investigation. **Xian Liu:** Investigation. **Chuanle Wang:** Investigation. **Shengcheng Deng:** Investigation. **Yue Yang:** Investigation. **Yuanyan Xiong:** Supervision. **Hongfei Xiang:** Investigation. **Jiyu Li:** Investigation. **Songbo Li:** Investigation, Writing – review & editing. **Wenbin Ma:** Conceptualization, Funding acquisition, Supervision, Writing – review & editing. **Songyang Zhou:** Conceptualization, Funding acquisition, Supervision, Writing – review & editing.

## Declaration of competing interest

The authors declare that they have no known competing financial interests or personal relationships that could have appeared to influence the work reported in this paper.

## Appendix A Supplementary data

Multimedia component 1

Table 1 AMPKbeta-interacting proteins identified by a genome-wide BiFC screen.
