# Supplementary material for: Targeting ROAM1 with UDP-GlcNAc nanosheets selective activates lysosomal AMPK to resolve metabolic dysfunction-associated steatotic liver disease
Source: Bioact Mater. 2026 Jul 12;66:487–507. doi: 10.1016/j.bioactmat.2026.06.047 (PMC13382097; doi:10.1016/j.bioactmat.2026.06.047)
Supplement: Multimedia component 1 [file mmc1.docx]

**Supplementary Figures**


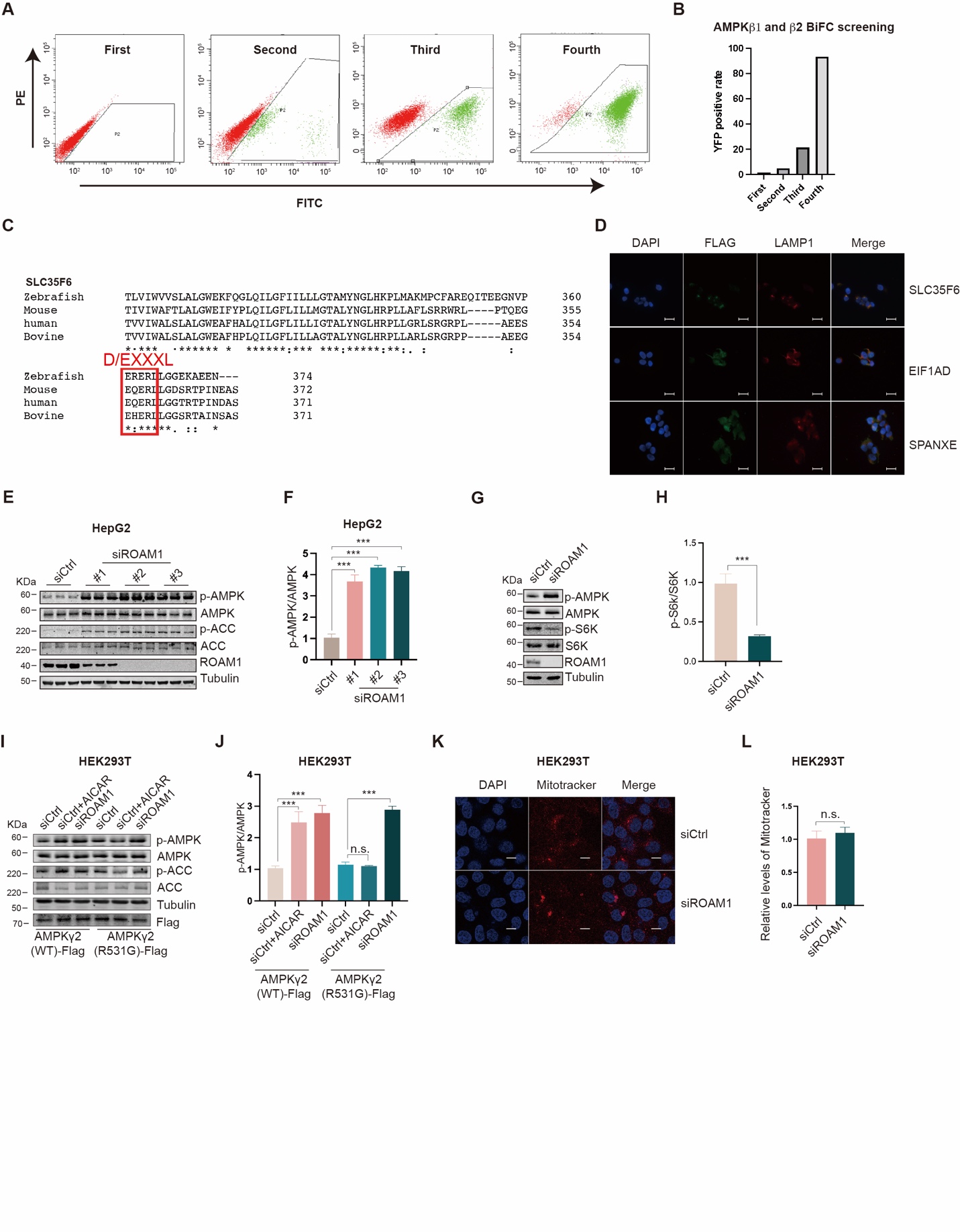


Supplementary Fig. S1

**(Α-Β)** HTC75 cells stably co-expressing YFPn-tagged AMPKβ1, AMPKβ2 and YFPc-tagged hORFeome library went through four rounds of FACS sorting and expansion in culture. Representative flow cytometry scatter plots (A) and percentages of YFP-positive cells (B) from each round are shown here. For gating, debris and dead cells were excluded based on low forward scatter (FSC) and side scatter (SSC). For BiFC assays, cells expressing YFPn-tagged AMPKβ1 or AMPKβ2 alone were used to define the YFP- population according to FITC/PE signals. A 30% PE to FITC compensation was applied.

**(C)** Sequence alignment of SLC35F6/ROAM1 from different species. The conserved canonical lysosomal sorting signal sequences are indicated.

**(D)** HEK293T cells expressing Flag-tagged EIF1AD, SPANXE, or SLC35F6/ROAM1 were co-stained with antibodies against the Flag epitope (green) and the lysosomal marker LAMP1 (red). DAPI was used to stain the nuclei (blue). Scale bar: 20 μm. EIF1AD and SPANXE are two candidate proteins from the BiFC screen and confirmed to co-IP with AMPK (data not shown).

**(E-F)** HepG2 cells expressing three different siRNAs against ROAM1 (siROAM1) were collected three days after transfection for WB analyses using the indicated antibodies (E) and quantification of p-AMPK levels (F). A scramble siRNA was used as control (siCtrl). Statistical analysis results are shown as mean ± SD; ***p < 0.001 by one-way ANOVA, n = 3.

**(G-H)** siCtrl and ROAM1-KD #3 cells from Fig. 1D were collected for WB analysis with the indicated antibodies (A) and p-S6K quantification (B). Error bars represent mean ± SD, n = 3. Statistical significance was calculated using the Student’s t-test. ***P < 0.001.

**(I-J)** HEK293T cells stably expressing wild-type (WT) AMPKγ2 or the AMP-insensitive mutant (R531G) were treated with siCtrl, siROAM1, or AICAR (1mM, 4 hours) as indicated. The cells were then collected for WB analyses using the indicated antibodies (I) and quantification of p-AMPK levels (J). Statistical analysis results are shown as mean ± SD; ***p < 0.001 by one-way ANOVA, n = 3. n.s., not significant.

**(K-L)** HEK293T cells transfected with siCtrl and siROAM1-#3 from Fig. 1D were stained with the MitoTracker Red FM dye (K) (Scale bar: 20 μm). Fluorescence signals were quantified and plotted in (L) as mean ± SD (n = 50). Statistical significance was calculated using the Student’s t-test. n.s., not significant.


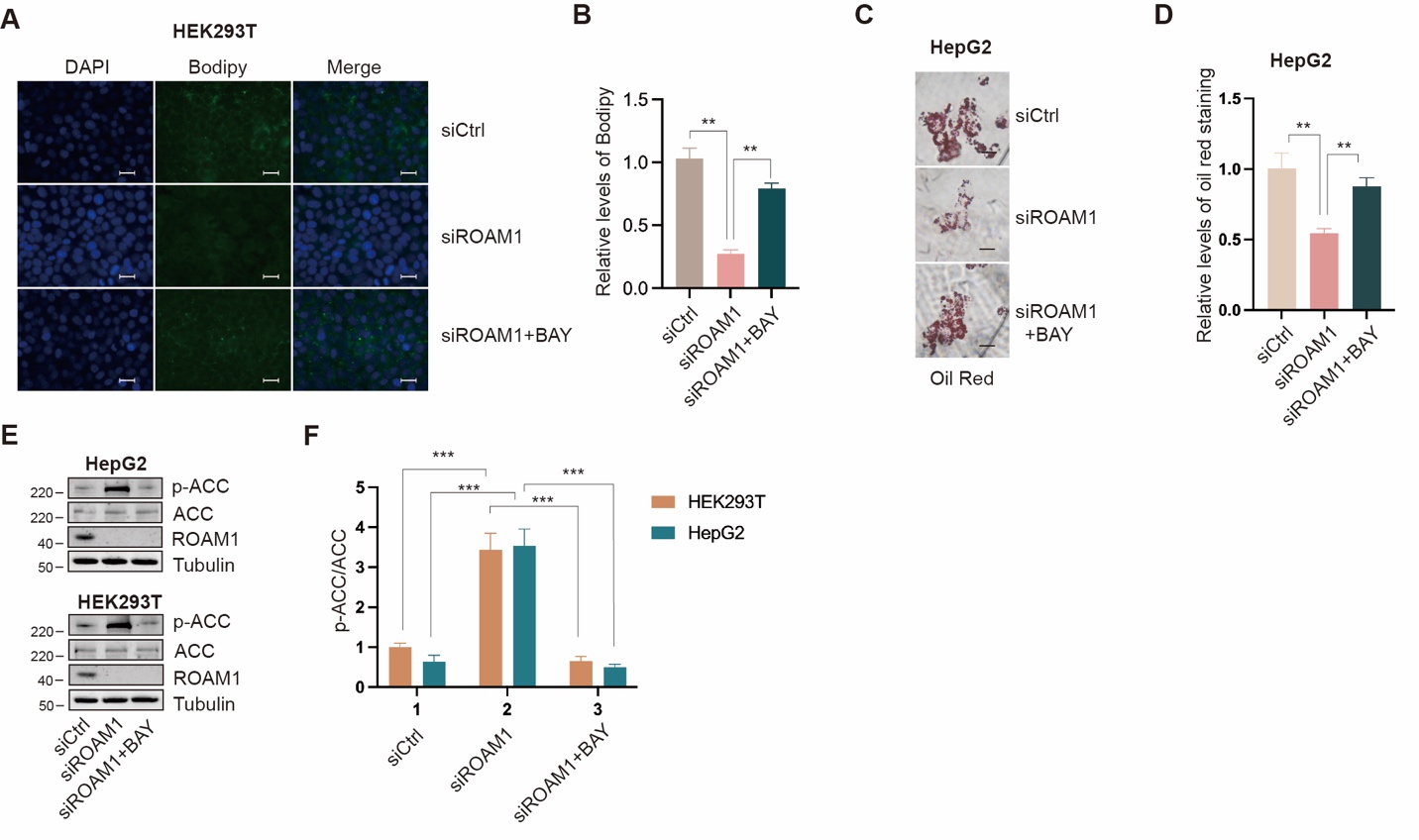


Supplementary Fig. S2

**(A-B)** Control and ROAM1 KD (siRNA#3) cells cultured with or without BAY-3827 (40μM) for 1h were stained with the BODIPY 493/503 dye (green) (A), which can stain neutral lipids and act as a tracer for oil and other nonpolar lipids. Hoechst 33342 (blue) was used to stain nuclear DNA. Scale bar: 5 μm. Fluorescence signals were quantified and plotted in (B) as mean ± SD (n = 3). Statistical significance was calculated using the Student’s t-test. **P < 0.01.

**(C-D)** ROAM1 KD (siRNA#3) HepG2 cells treated with or without BAY-3827 (40μM) for 1h were stained with Oil Red O (C), which can stain neutral lipids. Scale bar: 20 μm. Oil Red O signals were quantified and plotted in (D). Statistical analysis results are shown as mean ± SD; **p < 0.01 by one-way ANOVA, n = 50.

**(E-F)** ROAM1 KD (siRNA#3) HepG2 and HEK293T cells were treated with BAY-3827 (40μM) for 1h and then harvested for WB as indicated (E) and p-ACC/ACC quantification (F). Statistical analysis results are shown as mean ± SD; ***p < 0.001 by one-way ANOVA, n = 3.


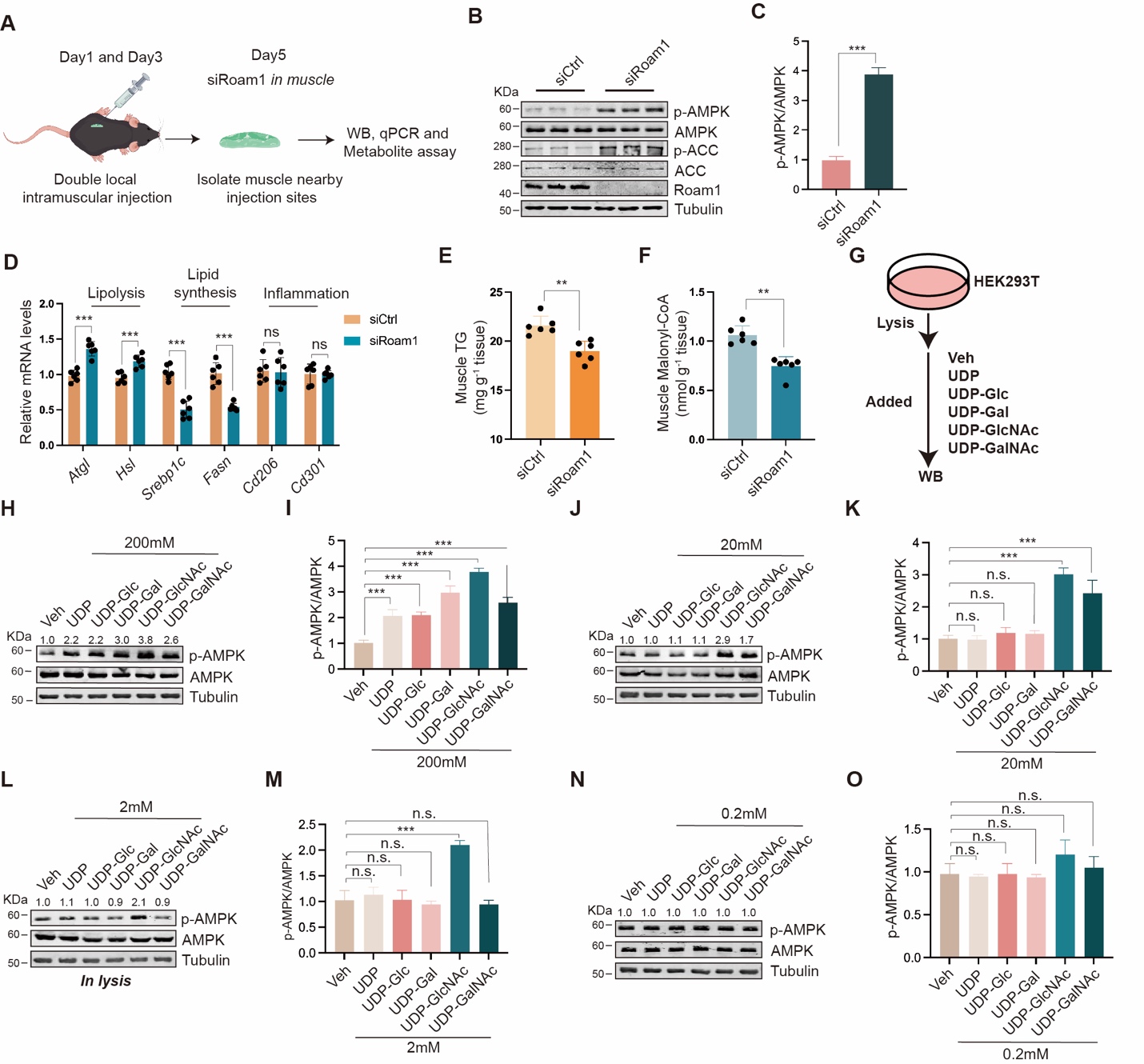


Supplementary Fig. S3

**(A-F)** For siRNA delivery into muscle (A), C57BL/6J mice received intramuscular injections of ROAM1-targeting siRNAs (4 nM) on days 1 and 3 in the same site. Muscle tissue near the injection site was collected on day 5 for WB (B) and p-AMPK quantification (C), qRT-PCR for genes involved in lipolysis, lipogenesis, and immune response (D), and triglycerides (TG) (E) and malonyl-CoA (F) levels. Statistical significance was calculated using the Student’s t-test; mean ± SD, n = 6; **P < 0.01, ***P < 0.001. siCtrl, a scramble siRNA.

**(G-O)** HEK293T cells pre-washed with low-salt n-Octyl beta-D-Glucopyranoside (ODG)-containing buffer were lysed in high-salt ODG buffer. The nonionic detergent ODG facilitates membrane protein solubilization. Various UDP-sugars were added to the lysates for one hour (G) at concentrations of 200mM (H), 20mM (J), 2mM (L)or 0.2mM (N) before WB analysis with the indicated antibodies. Quantification of p-AMPK levels is respectively presented in (I), (K), (M) and (O). Veh, 1xPBS. Statistical analysis results are shown as mean ± SD; ***p < 0.001 by one-way ANOVA, n = 3. n.s., not significant.


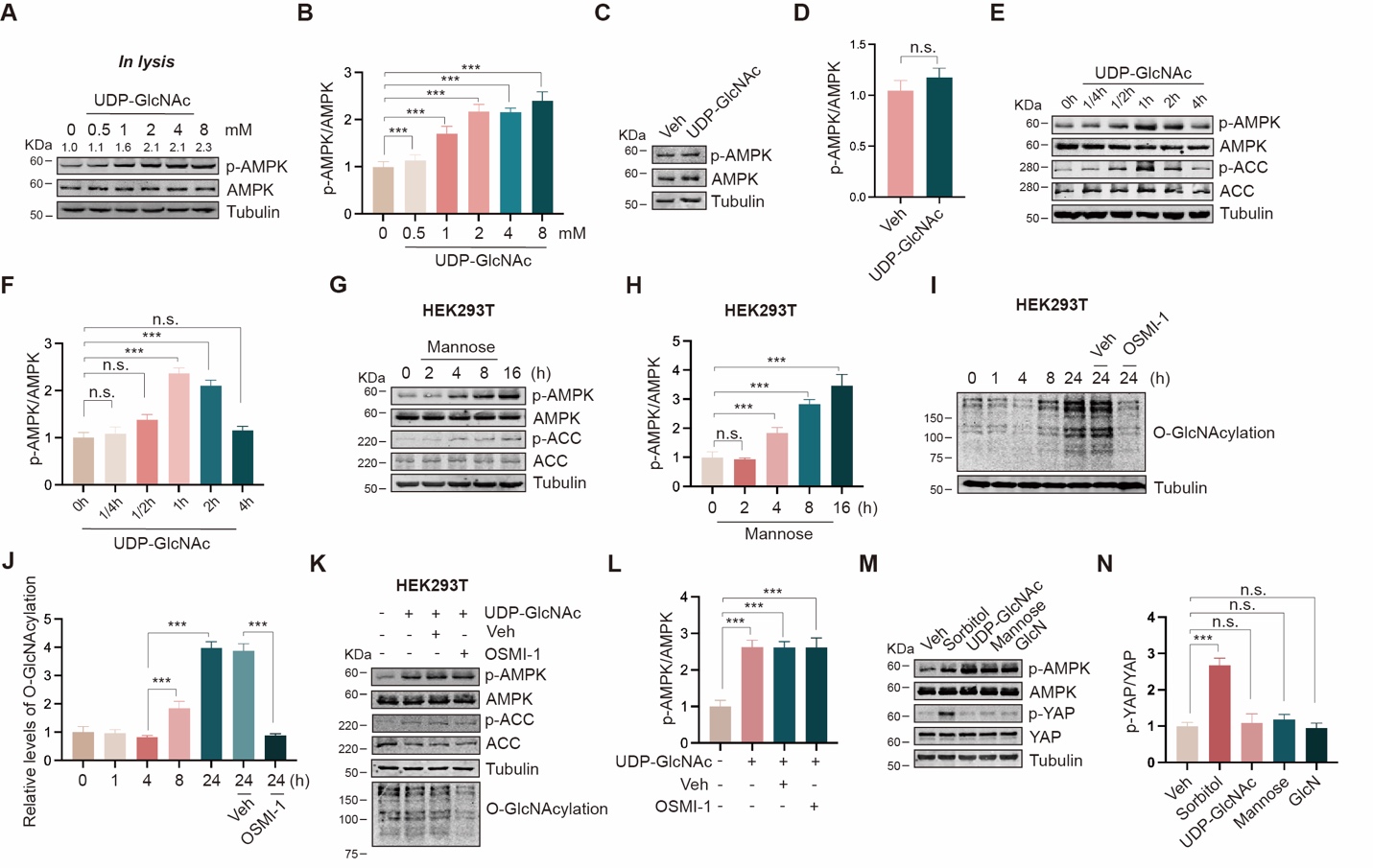


Supplementary Fig. S4

**(A-B)** The lysates of HEK293T cells were incubated with UDP-GlcNAc at the indicated concentrations for 1h before WB (A) and quantification of p-AMPK levels (B). Statistical analysis results are shown as mean ± SD; ***p < 0.001 by one-way ANOVA, n = 3. n.s., not significant.

**(C-D)** HEK293T cells cultured in the presence of UDP-GlcNAc (200mM) for one hour were lysed and analyzed by WB (C) and p-AMPK quantification (D). Error bars represent mean ± SD, n = 3. Statistical significance was calculated using the Student’s t-test. n.s., not significant. Veh, 1xPBS.

**(E-F)** HEK293T cells electroporated with UDP-GlcNAc (200 mM) were collected at the indicated time points for WB using the indicated antibodies (E). The levels of p-AMPK were quantified in (F). Statistical analysis results are shown as mean ± SD; ***p < 0.001 by one-way ANOVA, n = 3. n.s., not significant.

**(G-H)** HEK293T cells treated with 10mM mannose for varying durations were harvested for WB as indicated (G) and p-AMPK quantification (H). Statistical analysis results are shown as mean ± SD; ***p < 0.001 by one-way ANOVA, n = 3. n.s., not significant.

**(I-J)** HEK293T cells electroporated with UDP-GlcNAc (200 mM) were cultured for varying durations and then harvested for WB (I) and p-AMPK quantification (J). For OSMI-1 treatment, cells cultured for 24 hours after electroporation were also treated with DMSO (Veh) or the OGT inhibitor OSMI-1 (20 μM) for an additional 24 hours. Statistical analysis results are shown as mean ± SD; ***p < 0.001 by one-way ANOVA, n = 3.

**(K-L)** HEK293T cells were incubated with OSMI-1 (20 μM) for 2 hours before electroporation with 200mM UDP-GlcNAc. These cells were collected for WB analysis using indicated antibodies (K) and quantification of p-AMPK levels (L). Statistical analysis results were shown in mean ± SD; ***p < 0.001 by one-way ANOVA, n = 3. Veh, DMSO.

**(M-N)** HEK293T cells individually treated with Sorbitl (300mM, 15min), UDP-GlcNAc (200mM electroporation, 1hr), mannose (10mM, 8hr), or GlcN (5mM, 4hr) were collected for WB using the indicated antibodies (M) and quantification of p-YAP levels (N). Statistical analysis results are shown as mean ± SD; ***p < 0.001 by one-way ANOVA, n = 3. n.s., not significant.


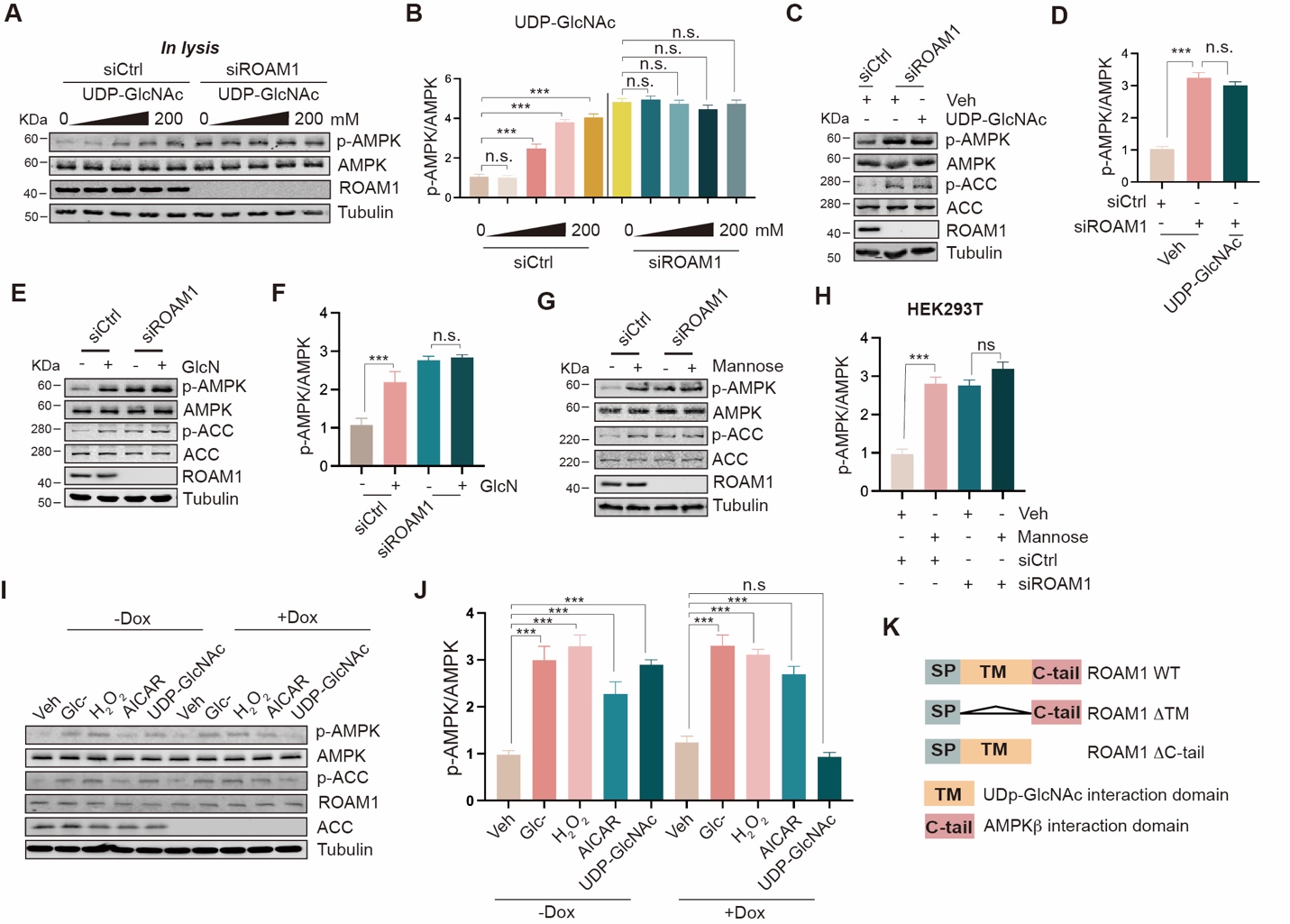


Supplementary Fig. S5

**(A-B)** Lysates from control (siCtrl) and ROAM1 KD (siRNA #3) HEK293T cells were incubated with increasing concentrations (0, 0.2, 2, 20, and 200mM) of UDP-GlcNAc for one hour before WB analysis with the indicated antibodies (A). p-AMPK/AMPK is quantified in (B). Statistical analysis results are shown as mean ± SD; ***p < 0.001 by one-way ANOVA, n = 3. n.s., not significant.

**(C-D)** Control (siCtrl) and ROAM1 KD HEK293T cells were electroporated with 200mM UDP-GlcNAc and then processed as in (Fig. 3D) for WB analysis with the indicate antibodies (C), and **q**uantification of p-AMPK levels in (D). Veh, 1xPBS. Error bars represent mean ± SD, n = 3. Statistical significance was calculated using one-way ANOVA. ***P < 0.001. n.s., not significant.

**(E-F)** Control and ROAM1 KD (siRNA#3) cells were harvested at 4h after GlcN (5mM) treatment for WB (E) and quantification of p-AMPK/AMPK (F). Statistical analysis results are shown as mean ± SD; ***p < 0.001 by one-way ANOVA, n = 3. n.s., not significant. Vehicle (1xPBS) alone served as negative controls.

**(G-H)** Control and ROAM1 KD (siRNA#3) cells treated with 10mM mannose for 8 hrs were collected for WB analysis with indicated antibodies (G) and quantification of p-AMPK levels (H). Veh, 1xPBS. Statistical analysis results are shown as mean ± SD; ***p < 0.001 by one-way ANOVA, n = 3. n.s., not significant.

**(I-J)** ROAM1 inducible knockout cells (ROAM1-KO #2) from (Fig. 1I) were cultured in doxycycline and treated with 1xPBS (Veh), glucose starvation for 16hr (Glc-), H_2_O_2_ (400 µM, 15min), AICAR (1mM, 4hr), and UDP-GlcNAc (electroporation)(200 mM, 1h). Cell extracts were used for WB using the indicated antibodies (I). The levels of p-AMPK were quantified (J). Statistical analysis results are shown as mean ± SD; n.s., not significant; ***p < 0.001 by one-way ANOVA, n = 3.

**(K)** ROAM1 domain organization and deletion mutants. SP, signaling peptide. TM, transmembrane region. C-tail, the C-terminal disordered peptide.


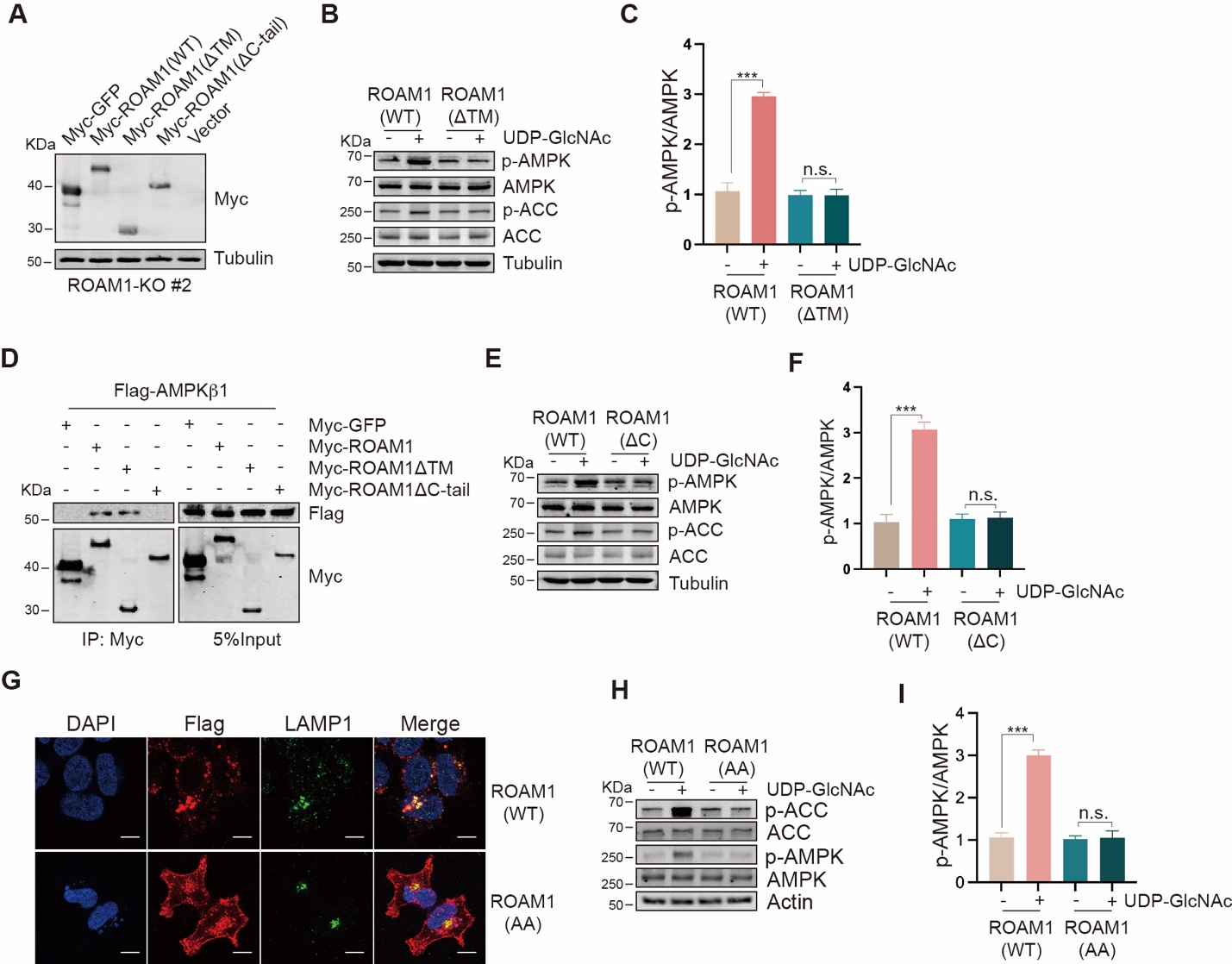


Supplementary Fig. S6

**(A-I)** ROAM1-KO #2 cells stably expressing Myc-tagged full-length (WT) or mutants (ΔTM, ΔC, CC) of ROAM1(L) were electroporated with UDP-GlcNAc (200 mM, 1h) or MgUGN (100 μg/ml) before WB as indicated (A, E, H). The levels of p-AMPK are quantified in (C, F, I). Statistical analysis results are shown as mean ± SD; n.s., not significant; ***p < 0.001 by one-way ANOVA, n = 3. Vehicle (1xPBS) alone served as negative controls.

**(D)** HEK293T cells co-expressing Flag-AMPKβ1 with Myc-tagged full-length or deletion mutants of ROAM1 were collected for anti-Myc IP and western blotting as indicated. Myc-GFP served as a negative control.

**(G)** HEK293T cells expressing Flag-tagged full-length (WT) or mutants (CC) of ROAM1 were were co-stained with antibodies against the Flag epitope (Red) and the lysosomal marker LAMP1 (Green). DAPI was used to stain the nuclei (blue). Scale bar: 5 μm.


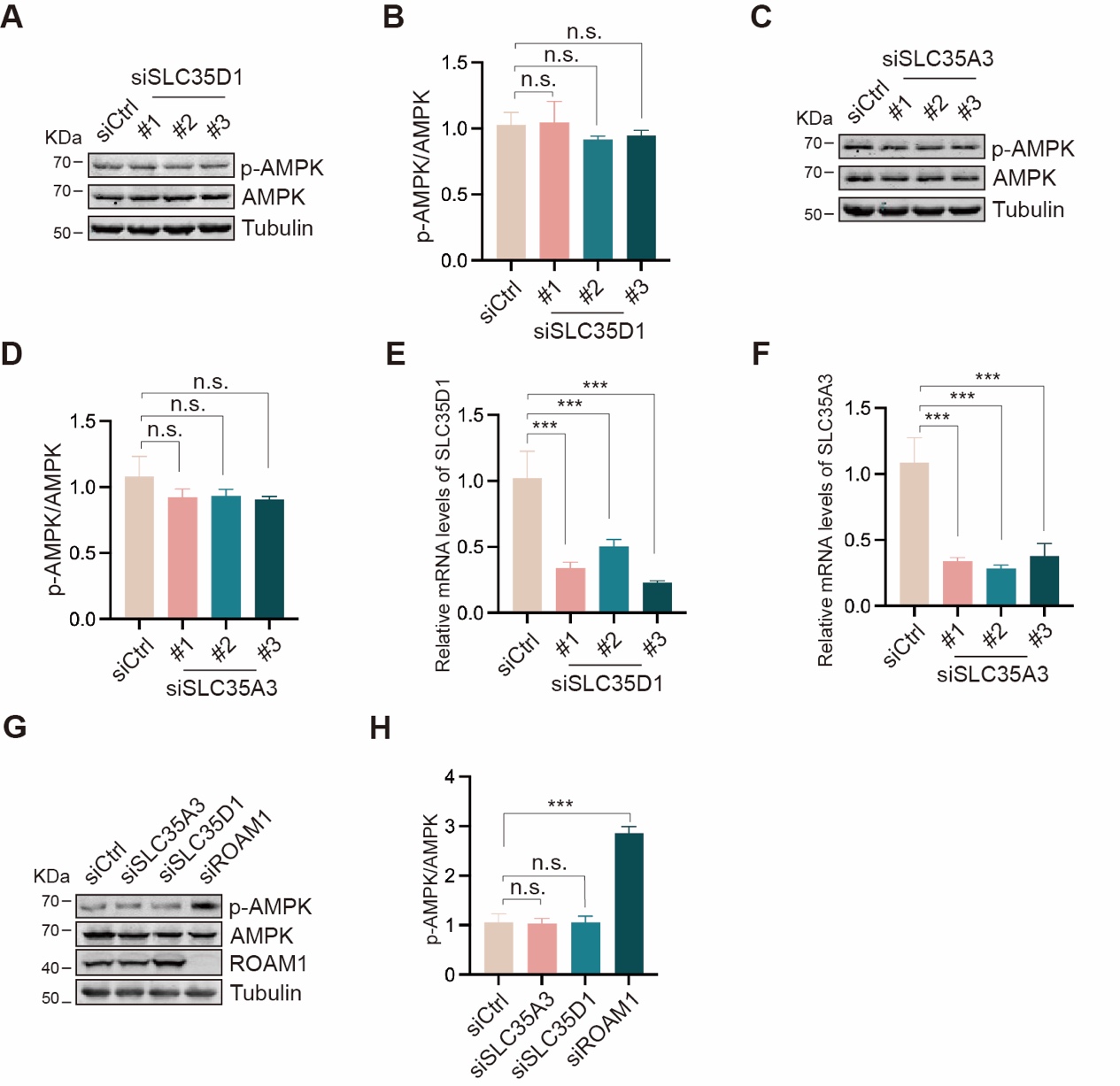


Supplementary Fig. S7

**(A-F)** HEK293T were treated with three different siRNAs against SLC35D1 (A) or SLC35A3 (C) for three days and processed for immunoblotting analyses as indicated. The levels of p-AMPK are respectively presented in (B) and (D). Relative changes in mRNA levels of SLC35D1 (E) and SLC35A3 (F) were determined by RT-qPCR. Error bars represent mean ± SD; n = 3 independent experiments. Statistical significance was calculated using one-way ANOVA. ***P < 0.001. n.s., not significant.

**(G-H)** HEK293T were transfected with control siRNAs (siCtrl) or siRNAs against SLC35A3 (#2), SLC35D1 (#3), or ROAM1 (#3) for three days, followed by immunoblotting analyses using the antibodies as indicated (G) and quantification of p-AMPK levels (H). Statistical analysis results are shown as mean ± SD; ***p < 0.001 by one-way ANOVA, n = 3. n.s., not significant.


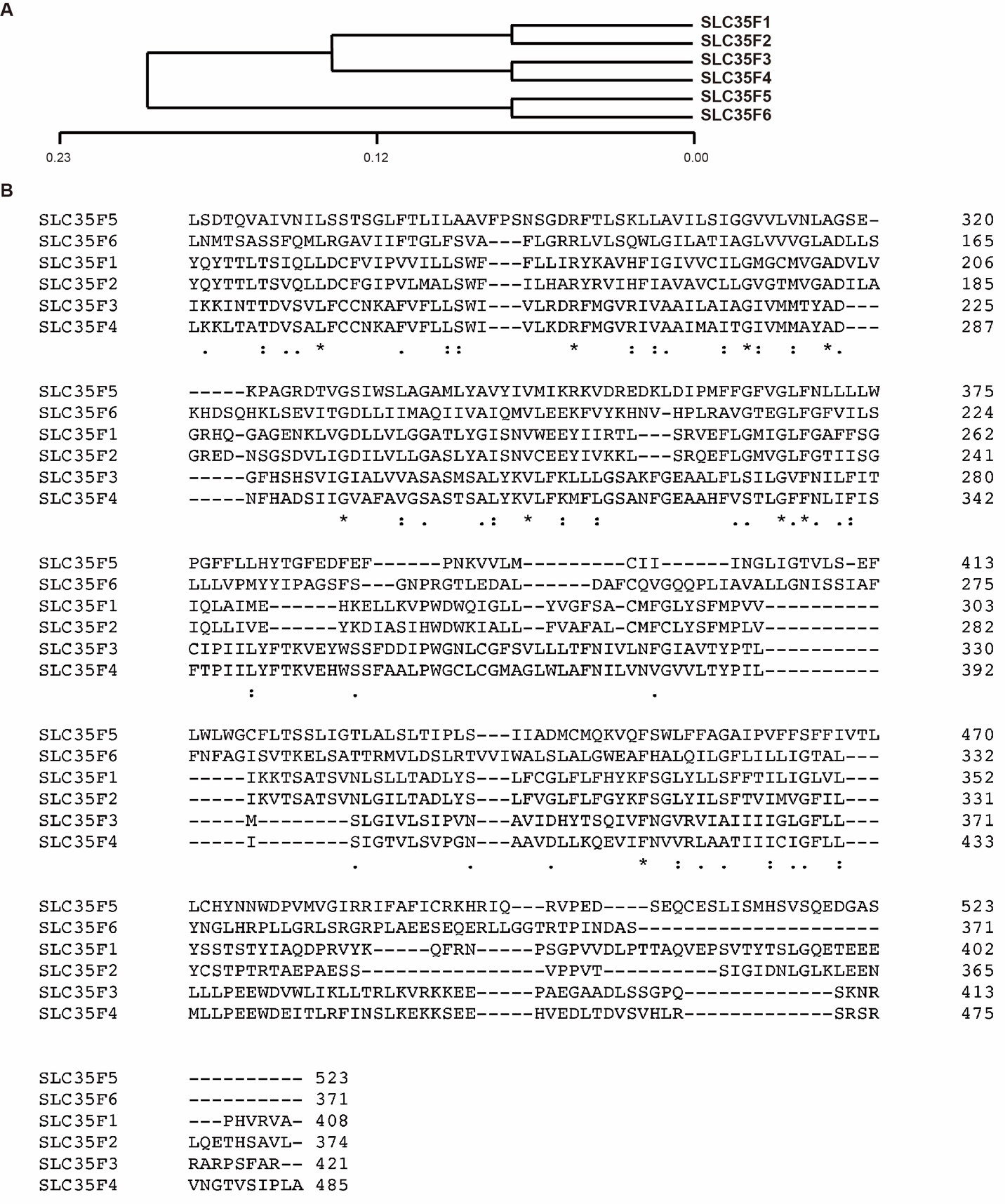


Supplementary Fig. S8

**(A)** The phylogenetic tree of the SLC35 family was generated by Discovery Studio 2.5. The SLC35F subfamily members included in this analysis were based on UniProt.

**(B)** Sequence alignment of human SLC35F subfamily proteins. Invariant residues are indicated by *. Closely related amino acids are indicated by : and ..


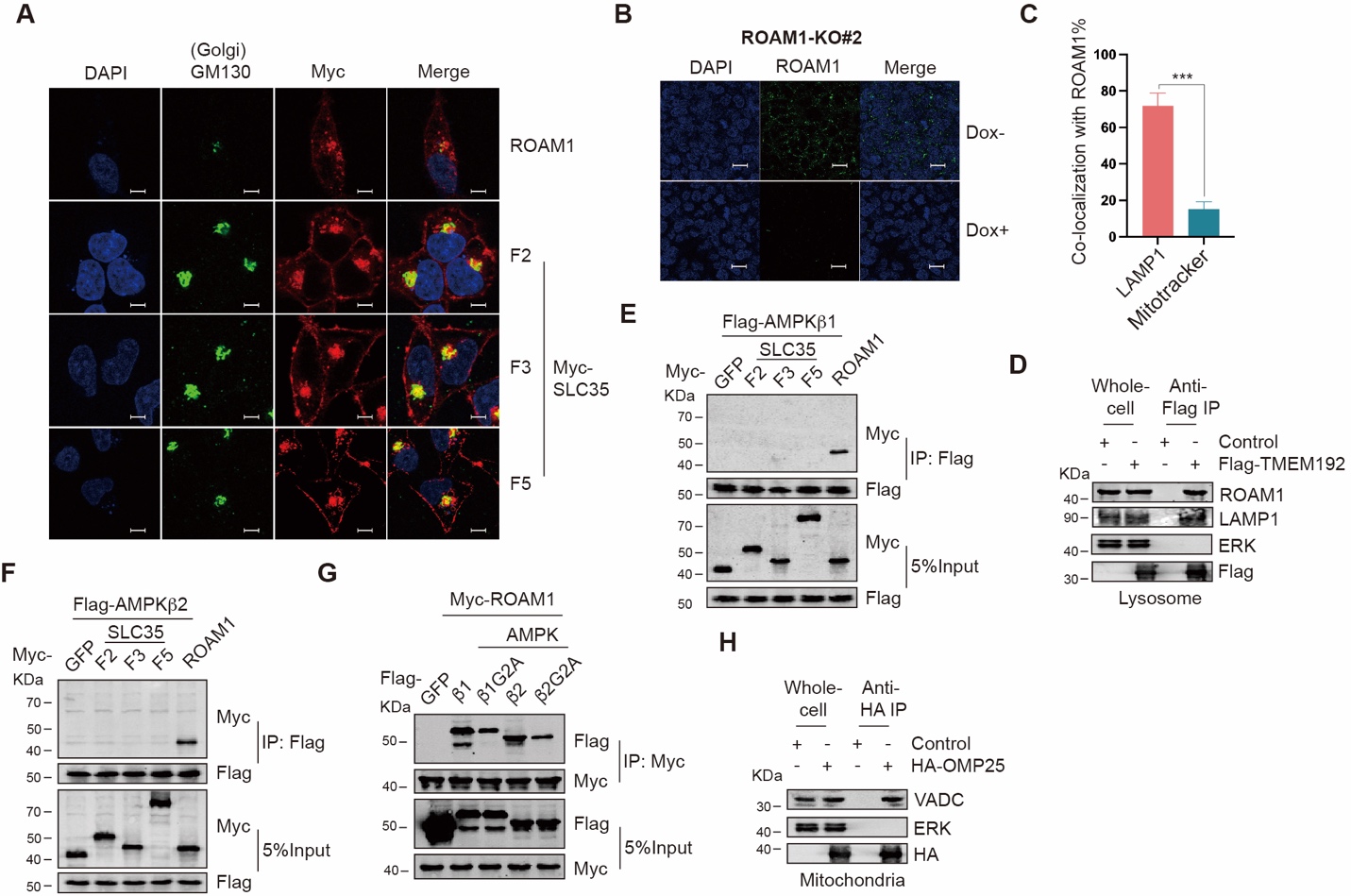


Supplementary Fig. S9

**(A)** HEK293T cells ectopically expressing the indicated Myc-tagged SLC35 family members were co-immunostained with antibodies against Myc (red) and the Golgi marker GM130 (green). DAPI was used to stain the nuclei (blue). Scale bar: 5 μm.

**(B)** Inducible ROAM1-KO#2 cells from Fig. 1I cultured with or without doxycycline were stained with an anti-ROAM1 antibody (Green). DAPI (blue) was used to stain nuclear DNA (Scale bar: 20 μm).

**(C)** Quantification of the co-localization between ROAM1 and LAMP1 or mitotracker. Error bars represent mean ± SD, n = 3. Statistical significance was calculated using the Student’s t-test. ***p < 0.001

**(D)** HEK293T cells expressing Flag-tagged TMEM192 were lysed with a Dounce homogenizer. Lysosomal fractions enriched by anti-Flag IP were analyzed by WB with the indicated antibodies. Vector alone was used as the negative control. The lysosomal marker LAMP1 and cytoplasmic protein ERK served as positive and negative controls respectively.

**(E-F)** HEK293T cells co-expressing the indicated Myc-tagged SLC35 family members with Flag-tagged AMPKβ1 (E) or AMPKβ2 (F) were collected for anti-Flag IP followed by WB as indicated. Myc-GFP served as a negative control.

**(G)** Myc-tagged ROAM1 was co-expressed with Flag-tagged AMPKβ1, AMPKβ1G2A mutant, AMPKβ2, or AMPKβ2G2A mutant in HEK293T cells. Cells were then harvested for IP with anti-Myc antibodies and western blotted as indicated. Flag-tagged GFP served as negative controls.

**(H)** HEK293T cells co-expressing HA-tagged OMP25 were lysed with a Dounce homogenizer. Mitochondrial fractions enriched by anti-HA IP were analyzed by WB with the indicated antibodies. Cells expressing vector alone served as a negative control. The mitochondrial marker VDAC and cytoplasmic protein ERK served as positive and negative controls for fractionation respectively.


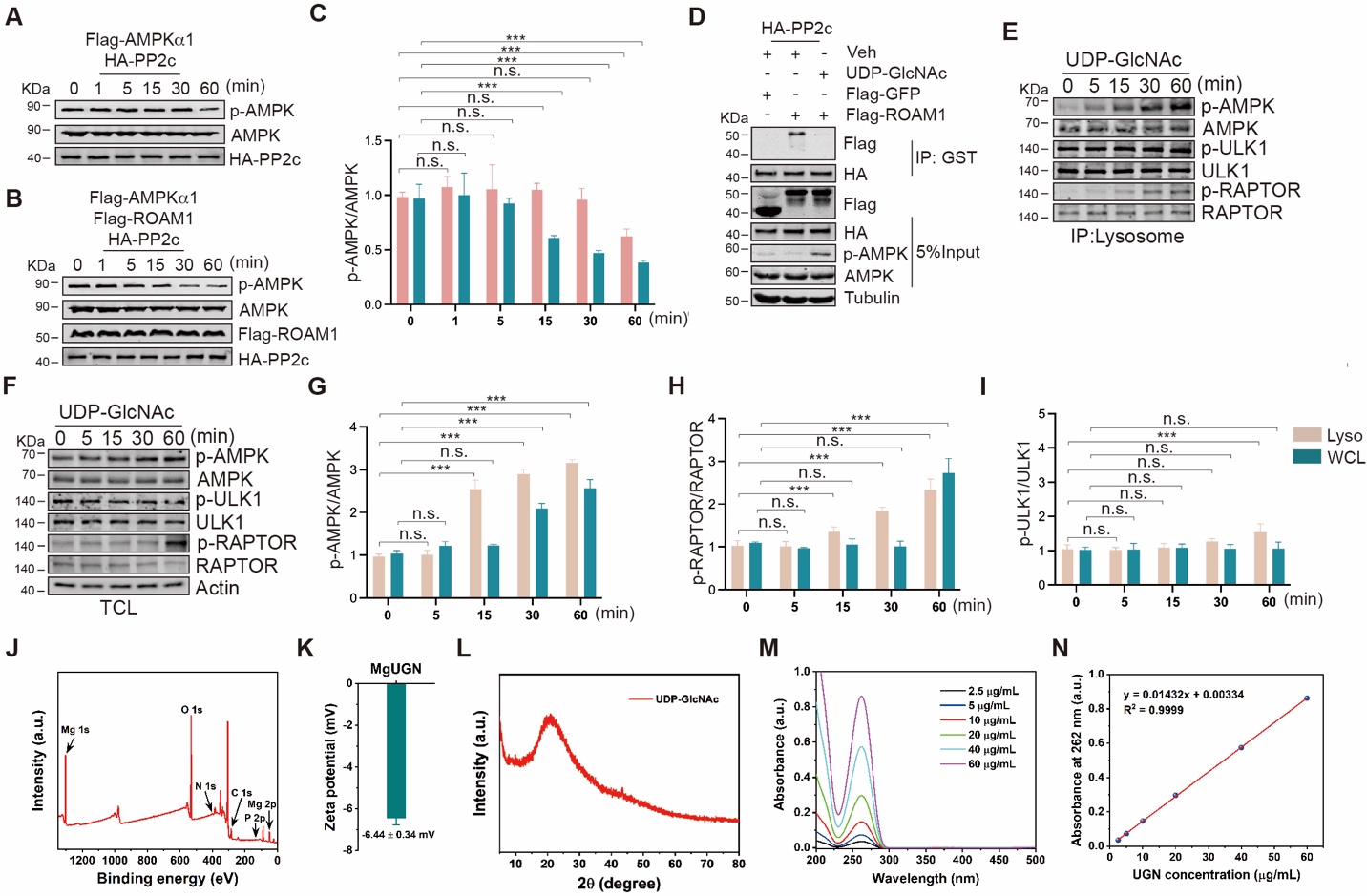


Supplementary Fig. S10

**(A-C)** HEK293T cells expressing Flag-AMPKα1 alone were treated with H_2_O_2_ for 5 mins to activate AMPKα1 and then collected for anti-Flag IP. HEK293T cells expressing Flag-ROAM1 or HA-PP2c were collected for Flag or HA beads pulldown. PP2c and AMPKα1 proteins mixed with (B) or without Flag-ROAM1(A) were then eluted and mixed together for in vitro dephosphorylation (37 ºC for one hour).(C) Statistical analysis results of p-AMPK/AMPK in (A, B) are shown as mean ± SD; ***p < 0.001 by one-way ANOVA, n = 3. ns, not significant.

**(D)** HEK293T cells co-expressing HA-PP2c with Flag-tagged AMPKβ1 were electroporated with UDP-GlcNAc (200mM) and then collected for HA beads pulldown and WB as indicated. Veh, 1xPBS.

**(E-I)** AML12 cells expressing Flag-tagged TMEM192 were electroporated with UDP-GlcNAc (200 mM) and cultured for the indicated times. Cells were then harvested for lysosome enrichment via anti-Flag IP, followed by Western blotting as shown in (E) and (F). (G, H, I) Quantification of p-AMPK (G), p-RAPTOR (H) or p-ULK1(I) levels in lysosomal and TCL, are presented as mean ± SD (n=3). ns, not significant; ***p < 0.001 by one-way ANOVA. Vehicle (1× PBS) alone served as negative control. TCL, Total-cell lysates.

**(J)** XPS survey scan of MgUGN nanosheets.

**(K)** Zeta potential of MgUGN nanosheets in aqueous solution. Data are presented as mean ± SD (n = 3).

**(L)** XRD pattern of pure UDP-GlcNAc.

**(M)** UV-Vis absorption spectra of aqueous solutions of UDP-GlcNAc at various concentrations.

**(N)** Absorbance at 262 nm exhibits a strong linear correlation with UDP-GlcNAc concentration.

**
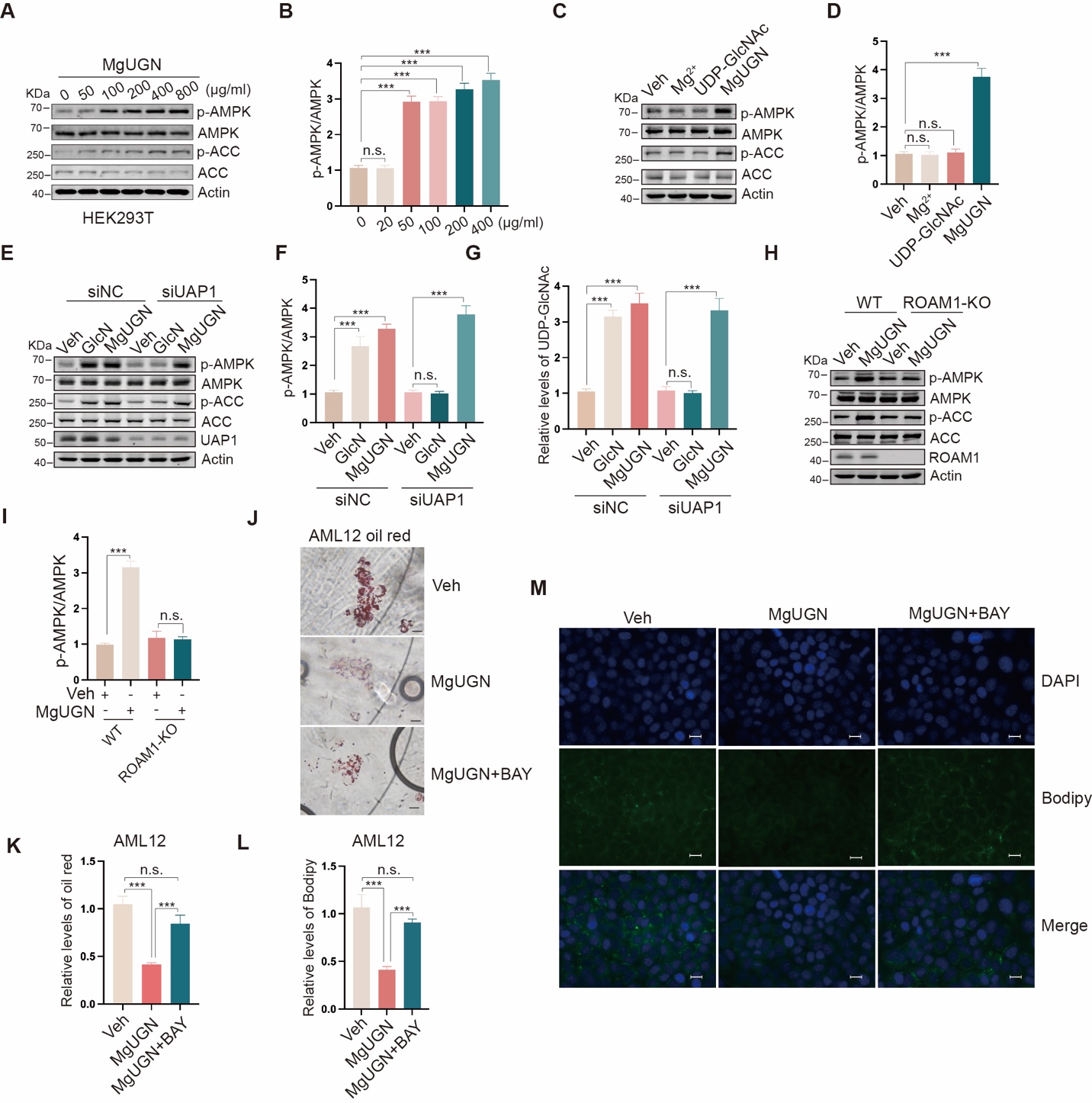
**

Supplementary Fig. S11

**(A-B)** HEK293T cells were cultured with different concentrations of MgUGN for one hour before being collected for WB analysis with the indicate antibodies (A). Quantification of p-AMPK levels is shown in (B). Vehicle (1xPBS) alone served as negative controls. Error bars represent mean ± SD, n = 3. Statistical significance was calculated using one-way ANOVA. ***P < 0.001. n.s., not significant.

**(C–D)** HEK293T cells were cultured with MgCl₂, UDP-GlcNAc, or MgUGN for one hour, followed by lysis and western blot analysis using the indicated antibodies (C). p-AMPK levels are quantified in (D) and shown as mean ± SD; ***p < 0.001 by one-way ANOVA, n = 3. Vehicle (Veh, 1×PBS) alone served as the negative control. n.s., not significant.

**(E-G)** HEK293T cells expressing UAP1siRNA#1 from Fig. 3N were treated with Veh (1xPBS), GlcN (5 mM), or MgUGN (100 μg/ml) for 1 h. Whole-cell lysates were used for western blot analysis with the indicated antibodies (E) and p-AMPK level quantification (F), followed by UDP-GlcNAc ELISA assay (G). Data are presented as mean ± SD, n=3. Statistical significance was determined by one-way ANOVA. ***P < 0.001; n.s., not significant.

**(H-I)** ROAM1-KO #2 cells from (Fig. 1I) cultured without Dox (WT) or with Dox (KO) were treated with 1xPBS (Veh) or MgUGN for 1 hour. Cell extracts were used for WB using the indicated antibodies (H). p-AMPK levels were quantified (I). Statistical analysis results are shown as mean ± SD; ***p < 0.001 by one-way ANOVA, n = 3. n.s., not significant.

**(J-K)** AML12 cells incubated with 1xPBS (Veh), MgUGN, or MgUGN plus BAY-3827 for 24h were stained with Oil Red O (J), which can stain neutral lipids (Scale bar: 20 μm). Oil Red O signals were quantified in (K). Statistical analysis results are shown as mean ± SD; ***p < 0.001 by one-way ANOVA, n = 50.

**(L-M)** AML12 cells treated with 1xPBS (Veh), MgUGN, or MgUGN plus BAY-3827 for 24h were stained with the BODIPY 493/503 dye (green) and Hoechst 33342 (blue). Fluorescence signals are quantified in (L) as mean ± SD (n = 3). Statistical significance was calculated using the one-way ANOVA. ***p < 0.001. Representative images are shown in (M). Scale bar: 10μm.


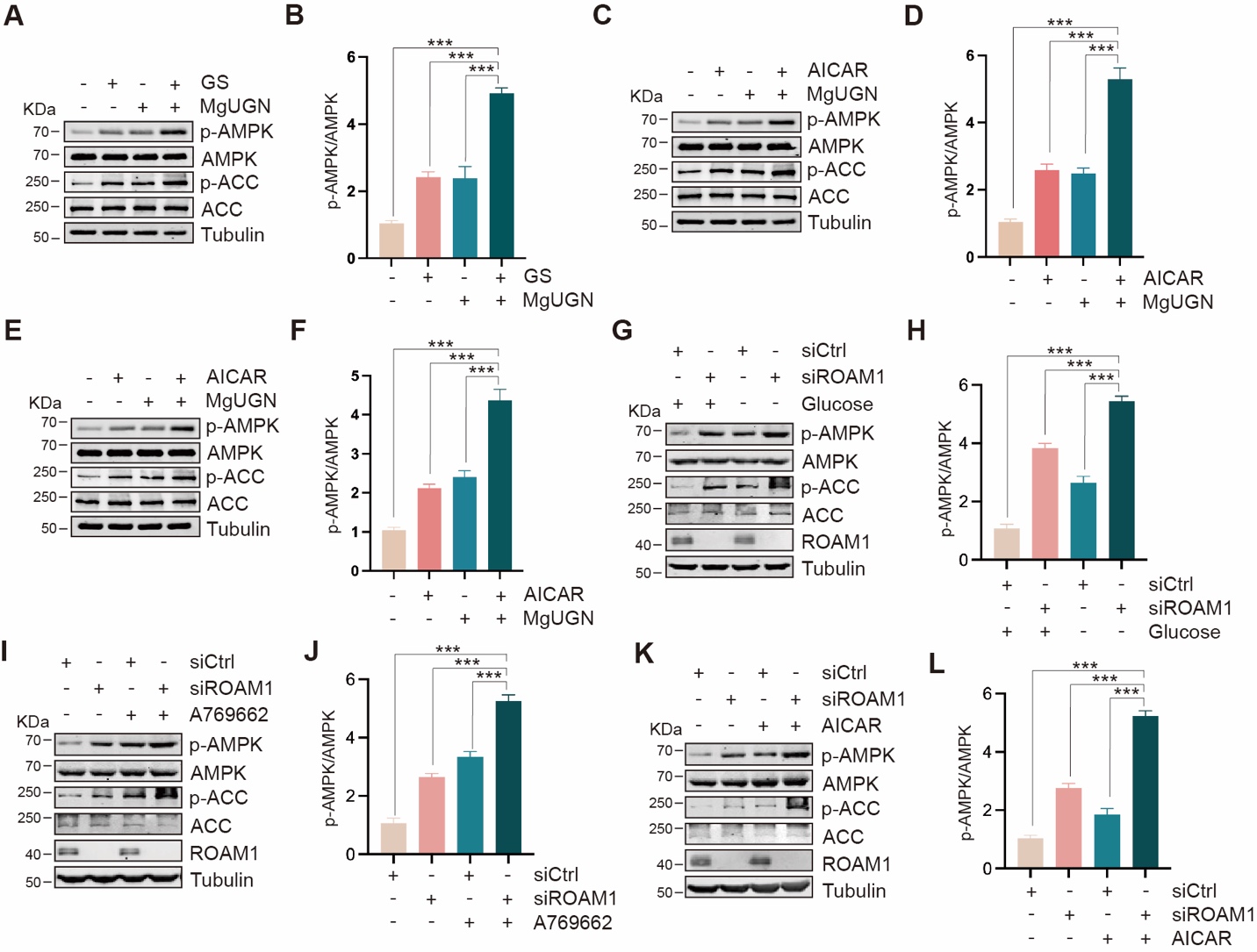


Supplementary Fig. S12

**(A-F)** AML12 cells were glucose starved (GS) for 16 hrs (A), or treated with 200μM A769662 for 4 hrs (C) or 1mM AICAR for 4 hrs (E), before being incubated with MgUGN (100 μg/ml) for 1 hour and analyzed by WB using the indicated antibodies. p-AMPK levels are quantified are respectively quantified in (B), (D), and (F). Statistical analysis results are shown as mean ± SD; ***p < 0.001 by one-way ANOVA, n = 3. 1xPBS treatment served as a negative control.

**(G-L)** Control (siCtrl) and ROAM1 KD (siRNA#3) cells were glucose starved for 16 hours (G), treated with 200μM A769662 for 4 hrs (I), or cultured with 1 mM AICAR for 4 hours (K) before WB analyses using the indicated antibodies. The ratios of p-AMPK/AMPK are respectively quantified in (H), (J), and (L). Statistical analysis results are shown as mean ± SD; ***p < 0.001 by one-way ANOVA, n = 3


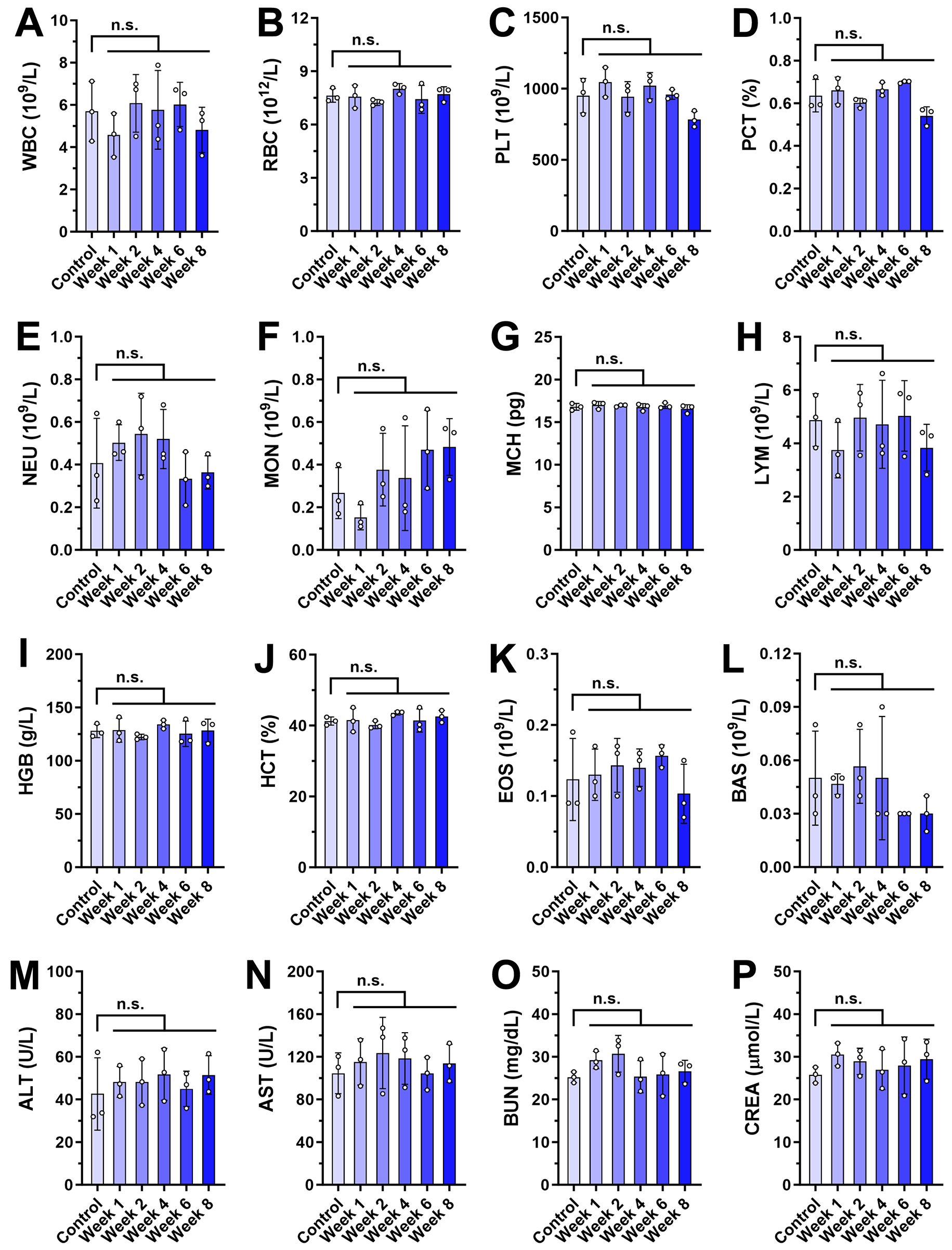


Supplementary Fig. S13

**(A-P)** Mice were intravenously administered MgUGN at a dose of 10 mg/kg body weight. Blood samples were collected at weeks 1, 2, 4, 6, and 8 post-injection. Hematological analysis of whole-blood parameters (A-L), and serum levels of alanine aminotransferase (ALT) (M), aspartate aminotransferase (AST) (N), blood urea nitrogen (BUN) (O), and creatinine (CREA) (P) are presented as mean ± SD (n = 3). Statistical analysis was performed using one-way ANOVA. n.s., not significant. Control, mice treated with PBS.


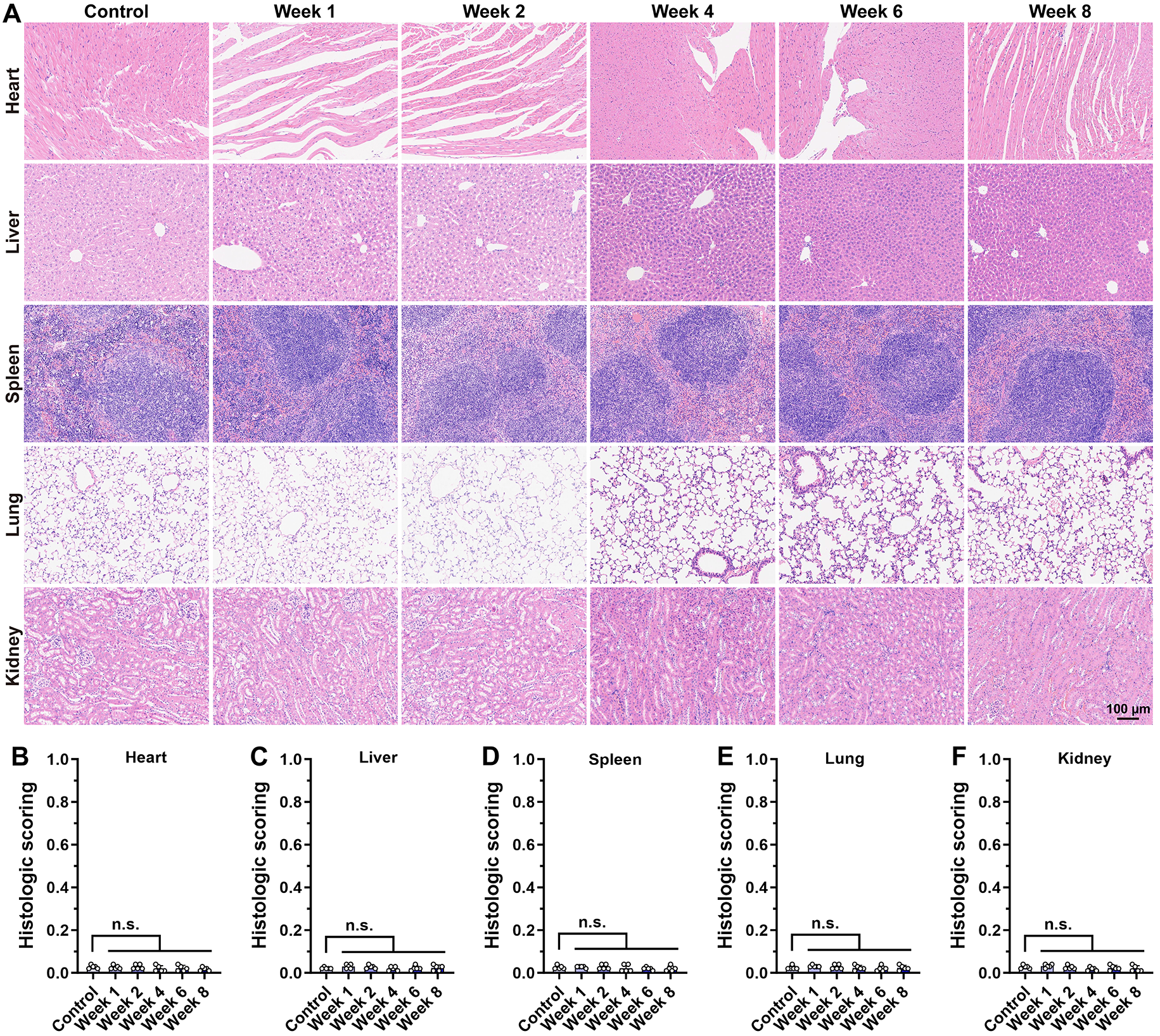


Supplementary Fig. S14

**(A-F)** Mice were intravenously administered MgUGN at a dose of 10 mg/kg body weight. Major organs were collected at weeks 1, 2, 4, 6, and 8 post-injection for H&E staining (A) and pathological scoring of the heart (B), liver (C), spleen (D), lung (E), and kidney (F). Statistical analysis was performed using one-way ANOVA. n.s., not significant. Control, mice treated with PBS.


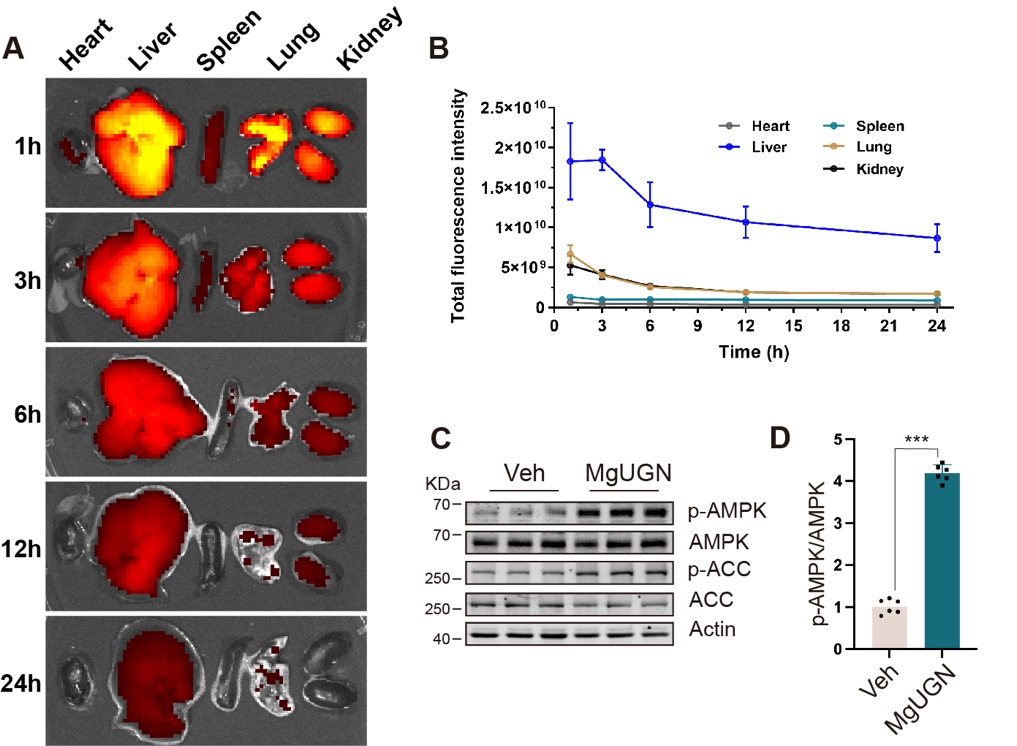


Supplementary Fig. S15

**(A-B)** Mice were intravenously administered Cy5.5-MgUGN at a dose of 10 mg/kg body weight. Major organs (heart, liver, spleen, lungs, and kidneys) were collected at the indicated timepoints post-injection for *ex vivo* fluorescence imaging (A) and fluorescence intensity quantification (B) (mean ± SD, n = 3).

**(C-D)** Eight-week-old male mice were administered MgUGN (10 mg/kg) via tail vein injection. After 48 hours, mouse liver tissues were collected for WB analysis using the indicated antibodies (C) and quantification of p-AMPK levels (D). Veh, 1xPBS. Error bars represent mean ± SD, n = 6 mice per condition. Statistical significance was calculated using the Student’s t-test. ***P < 0.001.

**
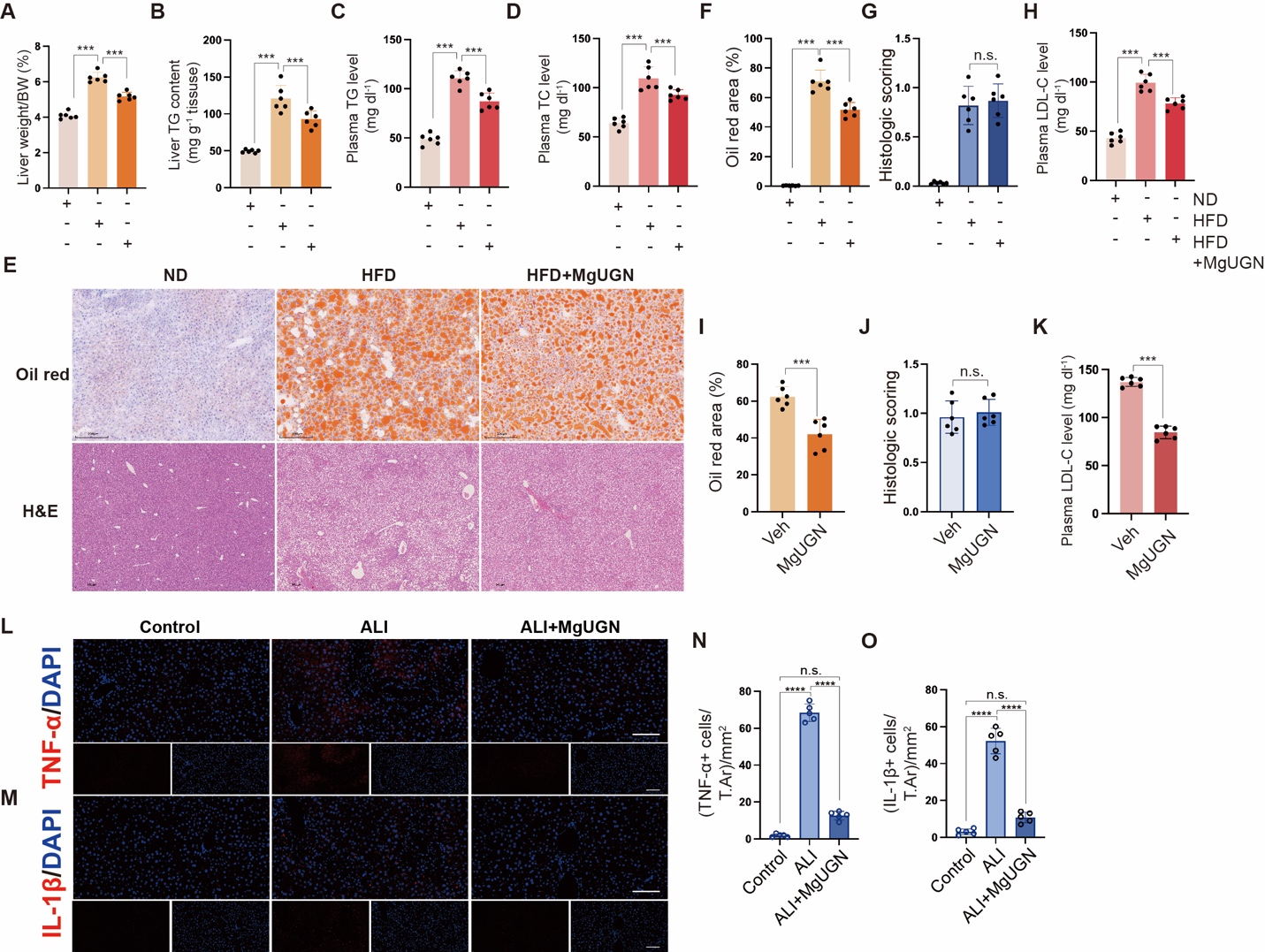
**

Supplementary Fig. S16

**(A-H)** Eight-week-old C57BL/6J male mice fed a high-fat diet (HFD) for 12 weeks were then administered MgUGN (10mg/kg) every 72 hours through tail vein injection for 14 days. Mice on a normal diet (ND) served as controls. Their liver to body weight ratio (A), hepatic (B) and plasma (C) TG levels, plasma TC (D) and LDL-C (H) levels. Liver sections were stained with H&E (Scale bar, 500 μm) and Oil Red O (Scale bar, 200 μm) (E), with Oild Red O quantification in (F) and H&E scoring in (G). Statistical analysis results are presented as mean ± SD; n = 6 mice per condition. ***p < 0.001 by one-way ANOVA, n.s., not significant.

**(I-J)** Quantification for Fig. 7A, for Oil Red O (I) and H&E (J) staining. Error bars represent mean ± SD, n = 6 mice per condition. Statistical significance was calculated using the Student’s t-test. ***P < 0.001. n.s., not significant.

**(K)** Eight-week-old *ob/ob* male mice were administered MgUGN every 72 hours through tail vein injection for 14 days. Plasma LDL-C levels are plotted as mean ± SD. ***p < 0.001 by Student's t-test, n = 6.

**(L-O)** Representative IHC images of TNF-α (L) and IL-1β (M) staining in liver tissue samples from healthy mice (control), APAP-induced liver injury mice (ALI), and ALI mice receiving MgUGN treatment (ALI + MgUGN). DAPI was used for nuclear staining. Scale bars: 100 μm. Quantification of TNFα+ (N) and IL-1β+ (O) cells is also presented. Error bars represent mean ± SD, n =5 mice per condition. Statistical significance was calculated using one-way ANOVA. ****p < 0.0001. n.s., not significant.
